# A lightweight and efficient rice field fertilizer applicator: structure, control, and test analysis

**DOI:** 10.3389/fpls.2025.1687293

**Published:** 2025-11-06

**Authors:** Chengsai Fan, Shijun Wan, Jianfu Sun, Gaoming Xu, Ruiyin He

**Affiliations:** 1 Key Laboratory of Intelligent Agricultural Equipment of Jiangsu Province, College of Engineering, Nanjing Agricultural University, Nanjing, China; 2 College of Intelligent Manufacturing and Equipment, Jiangmen Polytechnic, Jiangmen, China

**Keywords:** fertilizer applicator, side-depth fertilization, centrifugal distribution, control system, paddy fields

## Abstract

Side-deep fertilization in paddy fields is key to improving nitrogen use efficiency (NUE) and reducing surface water pollution. However, conventional applicators are overly heavy and incompatible with paddy machinery’s limited horsepower, restricting the technology’s popularization. To solve this, this study had two core goals: develop a lightweight, low-power centrifugal distribution-type side-deep fertilizer applicator matching paddy machinery’s load and horsepower limits; design a dedicated control system to enhance fertilization uniformity and fertilizer adaptability. First, the bulk density-based fertilizer model was improved through theoretical analysis of the external grooved wheel fertilizer discharging device, and its performance was validated in bench experiments. Simultaneously, the centrifugal distribution principle was analyzed, with dispenser rotational speed and discharge rotational speed selected as key factors and uniformity across rows chosen as the response variable. The optimum rotational speed-matching and optimal speed-matching models of the dispenser and distributor were established through CCD testing. The control system of the overall machine integrated both the bulk density-based fertilizer discharge and optimal speed matching models and performed tests in the field. The results showed that the average error in total fertilizer discharge is 4.84%, with a maximum error value of 7.93%, the average coefficient of variation for fertilizer discharge across rows was 5.47%, with a maximum coefficient of variation of 7.03%. Furthermore, comparative analyses revealed that the control system adapted well to different fertilizers and maintained stability between static and dynamic tests, thereby indicating strong dynamic adaptability. Compared with other fertilizer applicators for paddy field machinery, this device offers evident advantages in terms of quality, cost, and horsepower requirements, highlighting its potential for widespread adoption.

## 1 Introduction

Rice is the most important food crop in China, accounting for approximately 30% of the global total rice production. However, inappropriate fertilizer application in paddy cultivation causes water pollution and reduces economic efficiency (Su et al., 2025). The technology of synchronizing mechanical transplanting with side-deep fertilization is a major green technology. It involves placing slow-release fertilizer 3–5 cm to the side and 4–5 cm deep relative to the seedling roots. This placement improves nitrogen use efficiency (NUE), reduces environmental pollution, and can lead to savings of 10-20% in fertilizer use without yield loss (Feng et al., 2025; Guo et al., 2025; Yin et al., 2022). Therefore, rice side-deep fertilization technology is key to balancing the pollution of paddy fields while increasing grain yield and income (Shao et al., 2025). Despite its advantages, the integration of side-deep fertilization devices into paddy field planting machinery is complex. Paddy fields exhibit limited soil-carrying capacity and high forward resistance, necessitating a reduction in the weight of paddy seedlings or rice transplanters to allocate sufficient horsepower for trenching resistance in side-deep fertilization.

Existing fertilizer application devices feature a variety of different mechanical structures and can simply be classified into two types: one unit per row and one unit for multiple rows. one unit per row fertilizer applicators are commonly used in dry seeding (Wang, J. et al., 2023a). However, their mechanical structures are complex (Sun et al., 2020), with numerous repetitive mechanical structures increasing overall mass (Zha et al., 2020). To address these limitations, one unit for multi-row fertilizer applicators have been developed, including pneumatic distribution systems (Wang et al., 2024b; Yuan et al., 2022), mechanical rows fertilization application systems, and pneumatic-assisted fertilizer delivery systems (Cao et al., 2024). Wang et al. (2024a) developed a pneumatic distribution seeder that facilitates uniform fertilizer distribution across sixteen rows in a single pass (Wang et al., 2024a), whereas Cheng et al. (2022) designed a pneumatic distribution chamber fertilizer discharge system that enables uniform fertilizer application across eight rows in one ware. Cao et al. (2024) designed a mechanical fertilizer discharge system utilizing pneumatic-assisted delivery, enabling simultaneous fertilizer application and seed sowing. However, both of these methods require a fan, which greatly increases the quality of the overall fertilizer application system and consumes more horsepower. Liu et al. (2020) designed a fertilizer application device with lightweight structure and mechanical distribution; however, the maximum stable fertilizer discharge rate of this device is 1.6 kg/min, which cannot meet the operational requirements of this study. Meanwhile, it has a complex mechanical structure, leading to high difficulty in processing and assembly. Overall, the current fertilizer discharge structure still has room for optimization.

Numerous studies have been conducted on the control of fertilizer application rates. Fertilizer application devices can be classified into open-loop, semi-closed-loop, and fully closed-loop control systems (Zhang et al., 2025). An open-loop control system is the simplest control system, which means that the control system lacks any feedback signals. In this system, control signals are transmitted unidirectionally from the controller to the actuator. A semi-closed-loop control system detects fertilizer discharge flow indirectly by measuring the rotational speed of the fertilizer discharge shaft, which is an open-loop control method for fertilizer application (Reyes et al., 2015). On the one hand, a closed-loop control system monitors the flow rate in the fertilizer discharge pipeline; however, due to differences in the physical properties of different fertilizers, replacing fertilizers requires recalibrating the sensor parameters or models. Existing studies have developed various flow sensors for measuring particle flow based on principles such as photoelectric (Karimi et al., 2019), capacitive (Jia et al., 2020), electromagnetic, microwave (Zoad et al., 2023), and Doppler (Huang et al., 2021) technologies. Nevertheless, these sensors are still immature and not yet suitable for large-scale promotion and application. On the other hand, a closed-loop control system monitors the remaining fertilizer amount in the fertilizer box. For instance, Yu et al. (2019) monitored the remaining mass in the fertilizer tank based on the Kalman filter algorithm and weight principle. However, in the initial stage of operation, the change in the weight of fertilizer in the tank is relatively small, and machine vibrations in the field operation environment have a significant impact on detection accuracy (Yu et al., 2019). Zhao et al. (2021) used LiDAR to measure the volume of fertilizer in the tank (Zhao et al., 2021), but this method is relatively costly. Therefore, the optimal and easy-to-apply form of the semi-closed-loop control system at present.

Based on the aforementioned research status, the design principles of a side-deep fertilizer application device can be summarized as follows: Paddy field planting machinery has limited power availability and is sensitive to weight; the adoption of multi-row single-unit fertilizer applicators can help reduce the overall weight of the machine; direct flow detection technologies and sensors are not yet mature, making semi-closed-loop control systems more feasible for practical application; and fertilizer discharge uniformity can be optimized through specific control strategies (Reyes et al., 2015; Wang, H. et al., 2023).

Based on the above design principles, this study will develop a mechanical centrifugal distribution type, one-unit six-row side-deep fertilization device, whose main components include quantitative fertilizer discharge via a grooved wheel and centrifugal distribution by a disc. The overall mass of the fertilizer application unit is anticipated to decrease greatly because of the absence of a blower. Subsequently, a PCB peripheral circuit was designed using the STC8H1K16-36I-LQFP32 main control chip. By analyzing the fertilizer discharge model and the motion trajectories of fertilizer particles on the disc, an Adaptive Flow Control Model (AFCM) based on bulk density was developed for fertilizer discharge to achieve quantitative fertilizer discharge, and an Optimal Speed Matching Module (OSMM) was designed for the distributor to realize inter-row uniformity. Finally, the fertilizer application uniformity of the centrifugal distribution side-deep fertilization device and the effectiveness of its control system will be verified in field tests.

## 2 Materials and methods

### 2.1 Alignment of control systems with mechanical structures and principles

**Figure 1:**
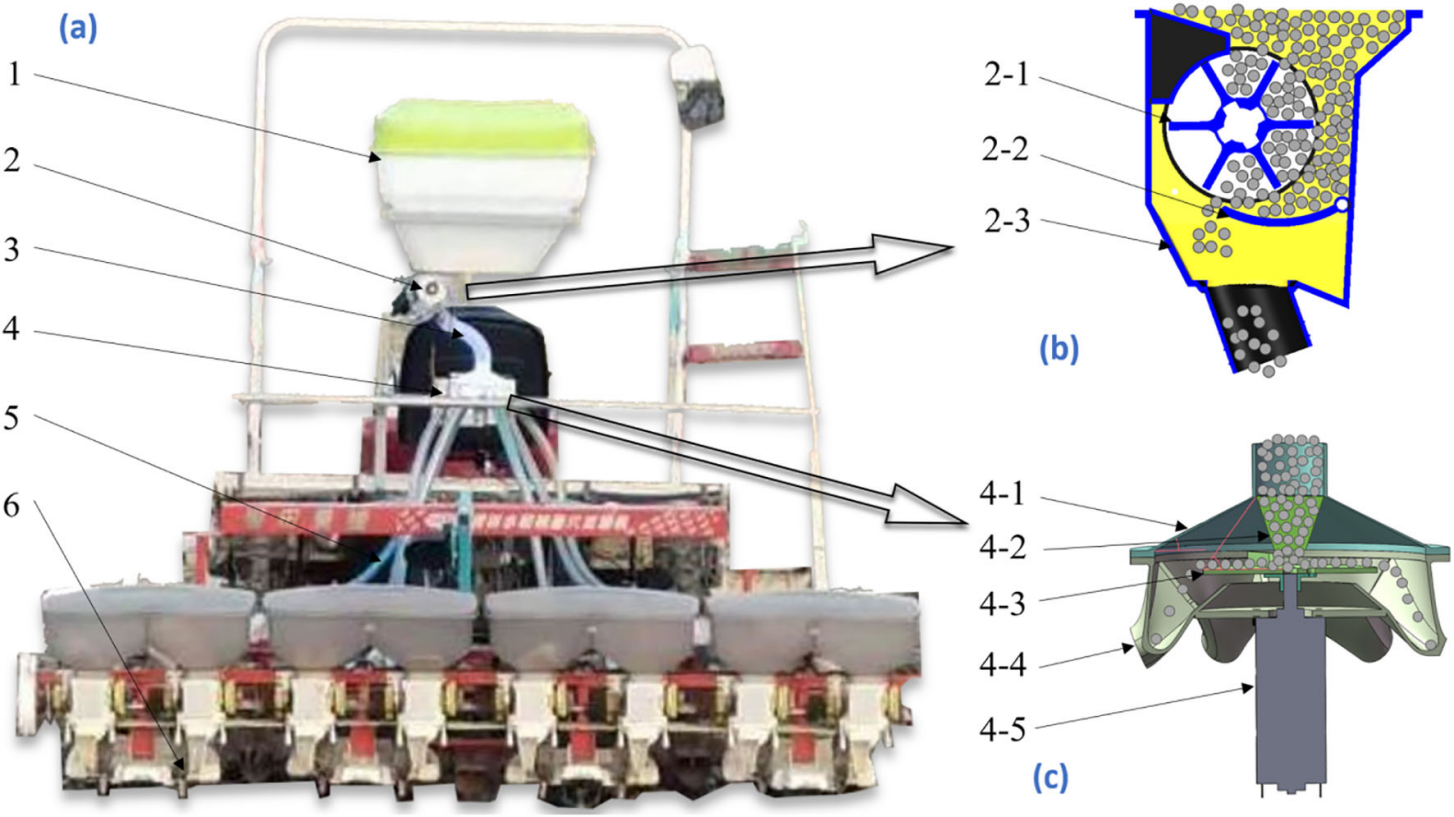
Overall structure of adaptive centrifugal distribution side deep fertilizer application device. **(A)** Overall structure. **(B)** Trough wheel type fertilizer discharger. **(C)** Distributor. 1 Fertilizer bin, 2 Trough wheel type fertilizer discharger, 3 Connecting tube, 4 Distributor, 5 Discharge tube, and 6 Trench opener.2–1 Trough wheel, 2–2 Fertilizer discharging tongue, and 2–3 Housing. 4–1 Distributor top cover, 4–2 Receiving hopper, 4–3 Centrifugal disc, 4–4 Distribution chamber, and 4–5 Motor.

The mechanically distributed fertilizer discharge system designed in this study was loaded onto a self-propelled rice transplanter as shown in **Figure 1A**. The system mainly consisted of a fertilizer bin, grooved-wheeled fertilizer discharger, connecting pipe, distributor, fertilizer discharge pipe, and furrow opener. The mechanically distributed fertilizer discharge system was positioned at the rear of the rice transplanter, while the furrow opener was staggered with the planting arm of the rice transplanter to realize side-deep fertilizer application. The fertilizer was quantitatively fed into the distributor from the connecting pipe through gravity and a groove wheel mechanism. It featured a disc, onto which the fertilizer fell to the center due to the collecting action of the funnel. The centrifugal force generated by the rotating disc evenly dispersed the fertilizer in all directions. It subsequently entered the fertilizer discharge pipe from the distributor outlet and finally fell into the furrow via the hollow structure of the furrow opener, thereby achieving side-deep fertilization.

### 2.2 Overall working principle of the control system

**Figure 2:**
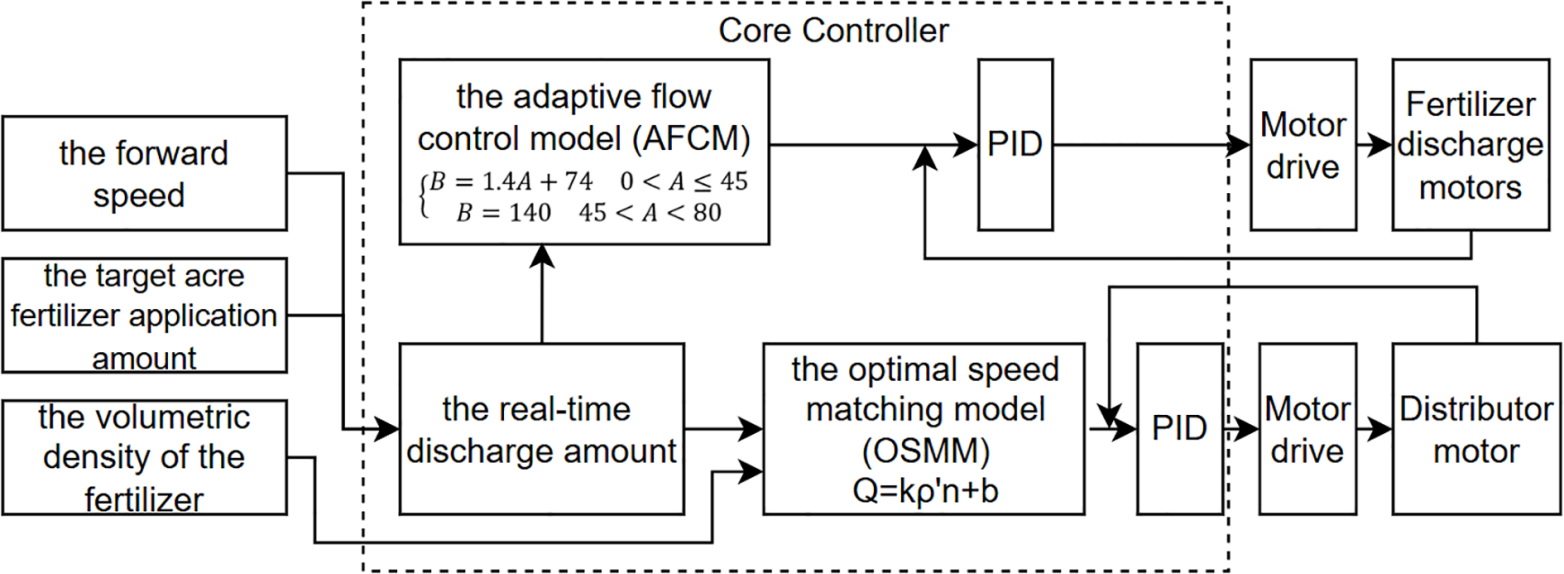
Control system schematic.

Prior to fertilizer applicator operation, the driver could adjust the target fertilizer application amount per acre using the knob on the controller. The Microcontroller Unit (MCU) controller calculated the target acre fertilizer application amount and forward speed of the fertilizer applicator as the inputs of the control system to derive the real-time required fertilizer discharge flow rate (Alameen et al., 2019). This flow rate and the fertilizer bulk density are inserted into the adaptive flow control model (AFCM) to calculate the fertilizer discharge shaft speed of the real-time discharge amount. Subsequently, the PID algorithm was employed to calculate the pulse width modulation (PWM) duty cycle for the control system output (Wang, H. et al., 2023). The real-time fertilizer discharge shaft speed control quantity was determined, and the required output PWM duty cycle of the control system was calculated using the PID algorithm (Wang, H. et al., 2023). The PWM signal was subsequently amplified by an opto-coupler isolated control drive circuit to control the real-time speed of the motor, thereby achieving precise control of the fertilizer application quantity. At the same time, the fertilizer discharge flow rate was input into the optimal speed matching model (OSMM) of the distributor to calculate the optimal distributor speed, while the PID algorithm was used to control the speed of the distributor in a closed loop. A control schematic for the synchronized fertilizer discharge control system is presented in **Figure 2**.

### 2.3 Embedded development board design

**Figure 3:**
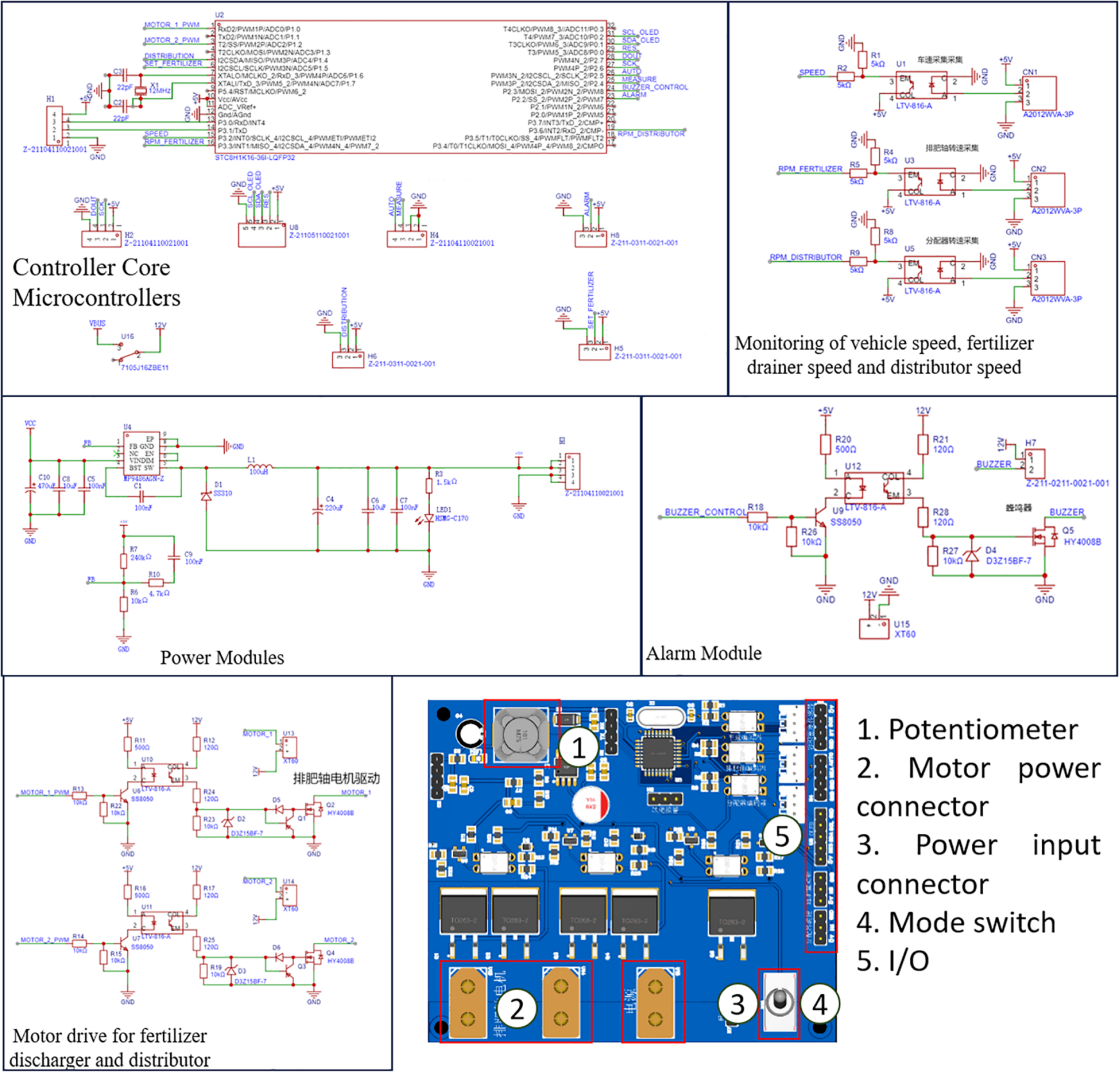
PCB circuit.

The PCB circuit board of the fertilizer discharge control system was designed with a highly integrated architecture. The board was equipped with an STC8H1K16-36I-LQFP32 main control chip configured with three independent three-pin I/O interfaces for connecting the rotary encoder. It facilitated the real-time acquisition of signals from the fertilizer shaft, distributor, and vehicle speed. Additionally, it incorporated an integrated H-bridge drive circuit to enable closed-loop control of both the fertilizer and distributor motors. Furthermore, it could be connected to the bulk density measurement module via the serial port. The power supply system innovatively adopted an aviation plug access program and incorporated a built-in DC-DC converter module, facilitating a dual-channel isolated power supply of 12V/5V, along with a Transient Voltage Suppressor (TVS) surge protection mechanism. The human-computer interaction module adopted an on-board welded encoder and Organic Light-Emitting Diode (OLED) screen I²C interface separation layout. This design ensured operational reliability and fulfils the assembly requirements of the control box. Additionally, integrating the signal conditioning circuit and three-proof coating process improves the anti-jamming ability of the fertilizer system and operational stability. The circuit is illustrated in **Figure 3**.

### 2.4 Control system hardware design

**Figure 4:**
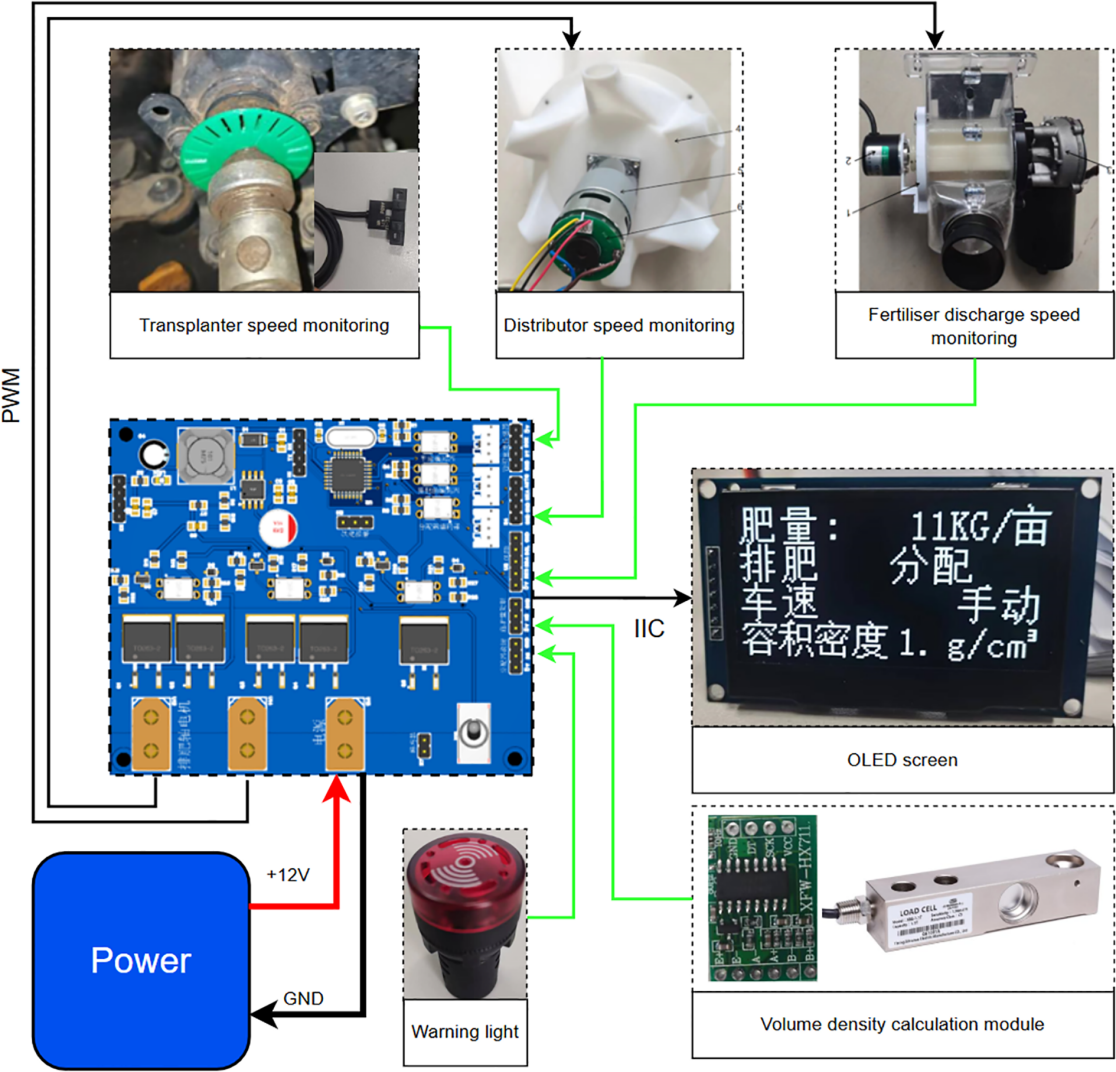
Hardware system setup.

The control system of the adaptive centrifugal distribution fertilizer applicator primarily consisted of nine modules: power output shaft speed measurement (vehicle speed measurement module), fertilizer discharge shaft speed measurement, distributor speed measurement, bulk density measurement, motor drive, alarm, human-computer interaction, display, and power supply modules as shown in **Figure 4**.

The velocimetry module collected rotational speed signals from three groups of independent encoders: fertilizer discharge shaft (GTS06-VE-RA400A-10M encoder, 400PPR), distributor (Hall sensor, 16PPR, M-method speed measurement), and rice transplanter power shaft (OMRON photoelectric switch, 22PPR, T-method speed measurement). The driver module adopted double parallel HY4008 MOS tubes (conduction internal resistance: 2.9 mΩ) to establish a bridgeless topology. This configuration operated in conjunction with the PID algorithm to realize closed-loop speed regulation for both the fertilizer discharging motor (0–80 rpm) and distributing motor (400 rpm). The power supply system incorporated an MP9486A wide-voltage converter (4.5–100V input), which supported compatibility with multiple types of lithium batteries and on-board power supplies, while assessing power supply stability under voltage fluctuation conditions. The bulk density detection module utilized the external YZC-133 high-precision load cell (± 0.05%) and a 500 ml standard container to facilitate volumetric dynamic calibration of fertilizer quality. Its removable design allowed for efficient measurement in the field. The system was optimized using 3D printing for grating components and protective structures were equipped with a drive shaft to prevent contamination in wet and decayed farmland environments. This demonstrated the high adaptability of the hardware design and agricultural scenarios.

### 2.5 Control system software design

#### 2.5.1 Main program design

**Figure 5:**
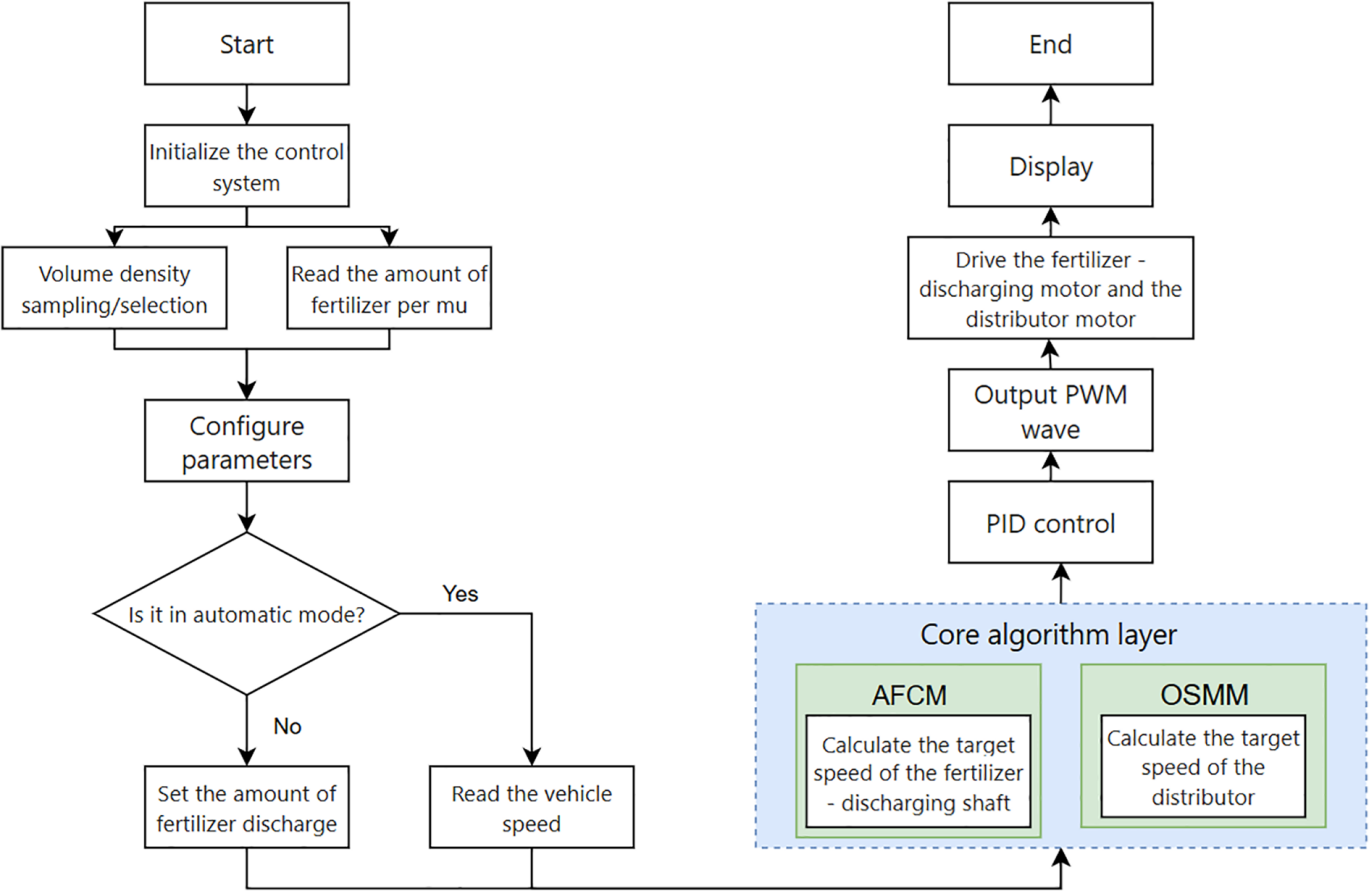
Control system main program.

The primary program of this fertilizer control system employed dual-mode interrupt-driven architecture to facilitate multitasking real-time scheduling via timer interrupts. As shown in **Figure 5**. In automatic mode, the system initially acquired the target acreage for fertilizer application and bulk density (measured or selected from saved data). The system subsequently triggered a 10 ms periodic interrupt based on the TIM1 timer from the STM32F4 family of microcontrollers for the purpose of vehicle speed acquisition (external pulse capture interrupt). Once the speed data were received, the adaptive control phase commenced, with the main loop persistently checking for updates from the host computer or changes in operating conditions. Concurrently, the core algorithmic layer adjusted the fertilizer discharge axle and distributor speeds in real-time. The core algorithm layer combined the AFCM and the OSMM, determining the target speed of the fertilizer discharge shaft/dispenser in real time via the floating-point operation unit (FPU). It triggered the PWM comparison interrupt of the TIM8 timer to achieve closed-loop PID adjustment, with an output duty cycle dynamic range of 0.1–99.9%. The manual mode did not require the collection of vehicle speed and operated solely based on the target amount of fertilizer discharge. It was primarily used as a backup mode for the automatic mode.

#### 2.5.2 Motor driver subroutine

The motor drive control relied on a TIM3/TIM4 timer to generate complementary PWM signals, which were isolated by a high-speed optocoupler (6N137) to drive double-parallel HY4008 MOS tubes. The gate charge acceleration circuit minimized the switching delay to approximately 18 ns. The characteristics of the DC brush motor included a design for dead time programmable adjustment (TIM_BDTR register configuration range of 96–158 ns). This was integrated with current sampling feedback (OPA2188 op-amp + 12-bit ADC synchronous sampling) to establish an overcurrent protection mechanism. When the detected peak was >8A immediately, the system promptly triggered DMA transmission to terminate PWM output. The PID regulator utilized an improved anti-integral saturation algorithm, employing the TPA2188 op-amp in conjunction with 12-bit ADC synchronous sampling. The PID regulator employed an improved anti-integral saturation algorithm and achieved a dynamic accuracy of ±2 rpm in speed error by triggering a 100 μs interrupt cycle via the TIM6 timer. This improved the response speed by 40% relative to that of the traditional PWM speed regulation scheme.

#### 2.5.3 Human-computer interaction subroutines

The human-computer interaction subroutine employed a bimodal input/output architecture design and incorporated a rotary encoder and an OLED display for dynamic parameter regulation and status visualization. The hardware layer recorded the user’s rotational speed setting value and operation command through the on-board welded encoder (resolution of 0.1°) and integrated these data with the 128 × 64 OLED screen connected through the I²C bus to display the fertilizer application amount, motor rotational speed, and system alarm information in real-time. At the software algorithm level, an event-driven mechanism was used to process the encoder pulse signals and key interrupts. An asynchronous response to operation commands (delay<10ms) was achieved through ring buffer management while optimizing the efficiency of screen refreshing based on the SSD1306 driver protocol to ensure that the visibility of the interface under the bright light environment in the field reached 300 cd/m ². The design enabled seamless transitions between the automatic and manual modes. Users could directly input target parameters via the rotary encoder. The system offered real-time feedback regarding control effects and equipment status, thereby establishing a closed-loop interaction link.

### 2.6 Adaptive flow control strategy design

The principle of fertilizer discharge for an external grooved wheel-type fertilizer discharger is illustrated in **Figure 1B**. The formula for calculating the discharge volume is as follows:

(1) $$Q=n\pi dL\rho \left(\frac{\alpha f}{t}+c\right)$$

where *Q* is the fertilizer discharge flow rate, g/min; *d* is the outer diameter of the outer groove wheel, cm; *L* is the effective working length of the groove wheel, cm; *ρ* is the mass per unit volume, g/cm^3^; *n* is the rotational speed of the fertilizer discharge shaft, rpm; *α* is the filling coefficient of the groove, which is obtained by experiment; *f* is the cross-sectional area of the individual grooves, cm^2^; *t* is the groove intercept distance of the groove wheel, cm; *c* is the driving force coefficient, cm.

(Equation 1) for calculating the discharge flow of the external grooved wheel fertilizer discharger indicates that the discharge flow is primarily influenced by variables *n*, *d*, *L*, *ρ*, *α*, *f*, *t*, and *c*. Notably, the parameters *d*, *L*, *f*, and *t* are determined by the mechanical structure of the fertilizer discharging mechanism. Within a reasonable range of rotational speed, *c* does not change much, *α* pertains to the shape of the fertilizer, while the displacement flow rate is predominantly influenced by the rotational speed n and bulk density *ρ*. The conventional displacement formula only quantitatively considers the rotational speed and bulk density.

A linear relationship is typically established between the rotational speed of the tank wheel and fertilizer discharge flow rate in the conventional model. The conventional discharge calibration equation is as follows (Equation 2):

(2) Q=kn+b

where *n* is the rotational speed of the fertilizer discharge shaft, rpm; *k* is the slope of the fitted curve; and *b* is the intercept of the fitted curve.

(Equation 2) is solely applicable to a specific type of fertilizer, and the error increases upon switching fertilizers; therefore, recalibration is necessary. Different fertilizer types have different bulk densities. We incorporated bulk density as an auxiliary parameter for fitting the linear primary term coefficients in the calibration formula and established a new calibration formula (Equation 3):

(3) $$Q=k\rho^{\prime }n+b$$

where *n* is the rotational speed of the fertilizer discharge shaft, rpm; *k* is the slope of the fitted curve; $\rho^{\prime }$ is the bulk density of the fertilizer; and *b* is the intercept of the fitted curve.

The optimized flow control model eliminates the need for calibration tests; however, it requires the input or measurement of the bulk density. The technical difficulty and time required for inputting or measuring the bulk density are much lower than those associated with the calibration test, rendering them nearly negligible in practical operations. AFCM was established based on (Equation 3), striving to enable the adaptation of various fertilizers within the model.

### 2.7 Distributor structure design and optimal speed matching strategy

#### 2.7.1 Distributor composition and principle of operation

The centrifugal distributor was mainly composed of an upper cover, centrifugal disc, flange, distribution chamber, and motor, as illustrated in **Figure 1C**.

During operation, the distributor released fertilizer from the outer groove wheel-type fertilizer discharger. Under the influence of gravity, the fertilizer entered the fertilizer inlet of the upper cover and descended to the center of the centrifugal disc. The centrifugal disc was mounted on the motor via the flange, with the motor facilitating the rotation of both the centrifugal disc and fertilizer. The centrifugal force caused the fertilizer to be ejected along the fan blades of the centrifugal disc in all directions to form a uniform fertilizer flow. When the fertilizer flow entered the distribution room, it was divided into six pathways to facilitate its delivery via the fertilizer delivery pipe for distribution.

#### 2.7.2 Kinematic analysis of fertilizer on a centrifugal disc

**Figure 6:**
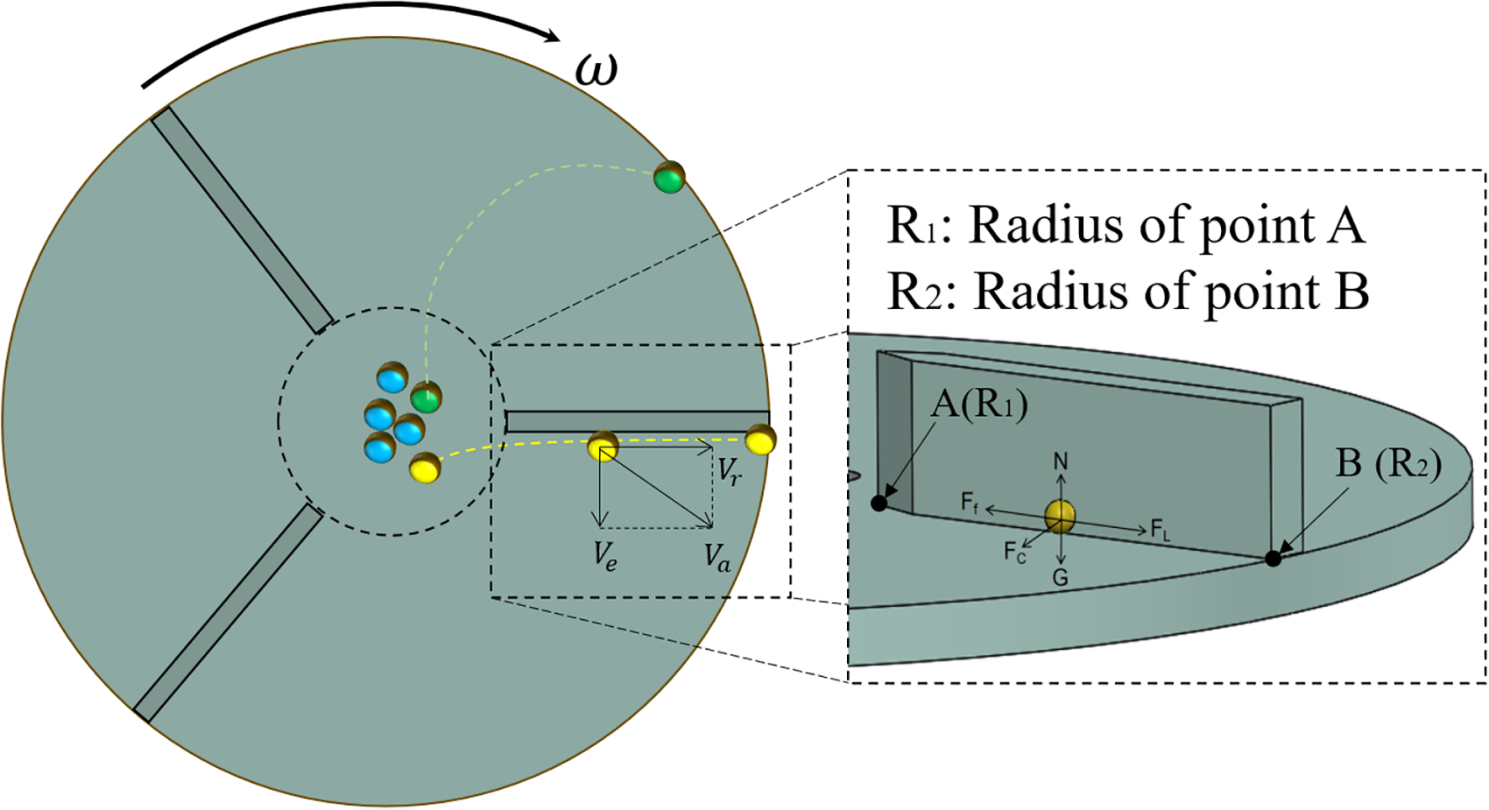
Force analysis of particles on the disc.

The fertilizer application disc dispersed fertilizer particles, which directly affected the degree of uniformity between the various fertilizer discharge ports. Most particles are thrown out by the fan blades and disc. The force analysis and motion analysis are shown in **Figure 6**.

The forces acting on the yellow part of the figure were analyzed, as illustrated in **Figure 6**.

Centrifugal force (Equation 4):

(4) $$F_{L}=m\omega^{2}r$$

Supporting force (Equation 5):

(5) N=G

Friction force (Equation 6):

(6) $$F_{f}=\mu N+\mu F_{C}$$

Support of fertilizer by fan blades (Equation 7):

(7) $$F_{C}=2m\omega Vr$$

Gravitational force (Equation 8):

(8) G=mg

where *m* is the mass of a single particle of fertilizer, kg; *ω* is the angular velocity of the centrifugal disc, rad/s; *r* is the radius of the centrifugal disc where the fertilizer is located, m; *μ* is the friction coefficient between the fertilizer and the centrifugal disc; *V_r_* is the relative velocity between the fertilizer and the centrifugal disc, m/s; *g* is the acceleration of gravity, m/s^2^.

The motion analysis of the fertilizer particles is presented in **Figure 6**. The fan blade on the centrifugal disc becomes perpendicular to the centrifugal disc, which serves as the reference coordinate system. The fertilizer particles exhibit liner motion on the centrifugal disc along the fan blade.

The relative motion of fertilizer particles (Equation 9):

(9) $$\frac{d^{2}x}{dt}+2\mu \omega \frac{dx}{dt}-\omega^{2}r=-\mu g$$

Its characteristic equation is as follows (Equation 10):

(10) $$\lambda^{2}+2\mu \omega r-\omega^{2}=0$$

The general interpretation is as follows (Equation 11):

(11) $$r=C_{1}e^{\lambda_{1}t}+C_{2}e^{\lambda_{2}t}+\frac{\mu g}{\omega^{2}}$$

Of which, The coefficients in Equation 11 are as shown in Equation 12.

(12) $$\left\{\begin{array}{c} \lambda_{1}=-\mu \omega +\sqrt{1+\mu^{2}}\omega \\ \lambda_{2}=-\mu \omega -\sqrt{1+\mu^{2}}\omega \\ C_{1}=\frac{\left(\sqrt{1+\mu^{2}}-\mu \right)\left(\mu g+R_{1}\omega^{2}\right)}{\sqrt{1+\mu^{2}}\left(1+\omega^{2}\right)+\mu \left(\omega^{2}-1\right)}-\frac{\mu g}{\omega^{2}}+R_{1}\\ C_{2}=\frac{\left(\mu -\sqrt{1+\mu^{2}}\right)\left(\mu g+R_{1}\omega^{2}\right)}{\sqrt{1+\mu^{2}}\left(1+\omega^{2}\right)+\mu \left(\omega^{2}-1\right)} \end{array}\right\}$$

The above kinematic analysis indicates that the motion of fertilizer on the fertilizer discharger is influenced by the friction coefficient (*μ*), the radius of point *A* (*R_1_*), and the angular velocity of the centrifugal disc (*ω*).

The friction coefficient ( μ) is a fixed parameter. Crucially, the radius at point A must be sufficient to ensure fertilizer lands on the disc, avoiding the fan blades. However, excessively increasing *R_1_* can lead to runaway accumulation of fertilizer particles, as exemplified by the green particles in **Figure 6**. Therefore, *R_1_* should be designed to be slightly larger than the diameter of the fertilizer outlet. The trajectory of the fertilizer particles is primarily governed by the rotational speed (ω). Therefore, Section 4.2 determines the optimal rotational speed of the disc under varying fertilizer flow rates through a full-factor test and develops the OSMM.

#### 2.7.3 Distribution room design

To determine the deflection angle α of the distribution chamber, the direction of the fertilizer’s absolute velocity *V_a_* must be identified. *V_r_* can be decomposed into *V_r_* and *V_e_*for separate calculations. The computation can be performed by neglecting the sliding friction force between fertilizer particles and the material.

The relative acceleration $a_{r}$ is given by (Equation 13), Expressed in differential form as shown in Equation 14:

(13) $$a_{r}=\omega^{2}x$$

(14) $$\frac{d^{2}x}{dt}=\frac{dv}{dt}=\frac{dv}{dx}\frac{dx}{dt}=\frac{dv}{dx}v=\omega^{2}x$$

after separating the variables and integrating both sides, we have (Equation 15):

(15) $$v^{2}=\int 2\omega^{2}xdx=\omega^{2}x^{2}+K$$

Assuming that when the fertilizer particle reaches point A on the inner edge of the centrifugal disc blade (where the radius is R_1_), the relative velocity is 0, we can substitute this into Equation 15 to obtain Equation 16:

(16) $$v=\sqrt{\omega^{2}x^{2}-\omega^{2}R_{1^{2}}}$$

Where $a_{r}$ is relative acceleration between the fertilizer particle and the centrifugal disc, m·s^-2^; ω is angular velocity of the centrifugal disc, rad/s, *x* is radial position of the fertilizer particle on the centrifugal disc, m; R_1_ is the radius of the inner ring of the centrifugal disc, m.

When the fertilizer leaves the centrifugal disc, substituting *x* = R_2_ into Equation 16 yields Equation 17. The convected velocity is as shown in Equation 18.

(17) $$v_{r}=\sqrt{\omega^{2}R_{2}^{2}-\omega^{2}R_{1}^{2}}$$

(18) $$v_{e}=\omega R_{2}$$

The angle α between the relative velocity *Vr* and the convected velocity *Ve* can be calculated by the Equation 19:

(19) $$\alpha =\arctan\frac{v_{e}}{v_{r}}=\frac{\omega R_{2}}{\sqrt{\omega^{2}R_{2}^{2}-\omega^{2}R_{1}^{2}}}\geq \frac{\pi }{2}$$

**Figure 7:**
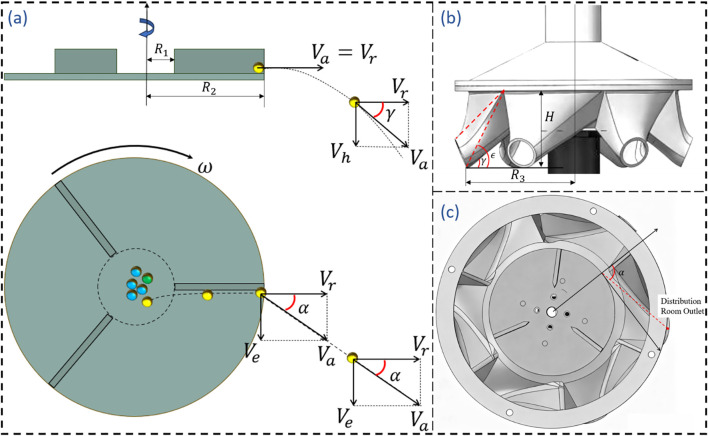
Distribution room design. **(A)** Kinematic analysis of fertilizer particles leaving the fertilizer disc. **(B)** Design of the drop angle (*γ* and ϵ) of the distribution chamber. **(C)** Design of the deflection angle ( α) of the distribution chamber.

Based on theoretical calculations and experiments, parameter α is set to 60°. As shown in **Figure 7C**.

The determination of the drop angle of the distribution chamber is guided by two main principles. First, to prevent fertilizer particles from accumulating, the angle between the outlet and the horizontal should be greater than the angle of repose of the fertilizer; here, we select γ = 45°. Second, it is preferable to ensure that most fertilizer particles can leave the distribution chamber without collisions. A kinematic analysis of particles leaving the disc is shown in **Figure 7A**. The distance from the outlet of the distribution chamber to the center of the disc is R3 as shown in **Figure 1**, so the distance from the disc is R_3_-R_2_.

The radial velocity is given by Equation 17, and since the vertical motion is uniformly accelerated, the following relationship can be established (Equations 20–22):

(20) $$R_{3}-R_{2}=v_{r}t$$

(21) $$H=1/2\times gt^{2}$$

(22) $$tan\epsilon =\frac{H}{R_{3}-R_{2}}$$

where, ${\text{R}}_{3}$ refers to the radial distance from the outlet of the distribution chamber to the center of the disc, with the unit of meters (m); H refers to the vertical distance from the outlet of the distribution chamber to the edge of the disc, with the unit of meters (m); ϵ refers to the angle between the line connecting the edge of the disc and the outlet and the horizontal direction, with the unit of degrees (°); and t refers to the time it takes for a fertilizer particle to travel from the disc to the outlet of the distribution chamber, with the unit of seconds (s).

With a radius of 4.8 cm and a rotational speed of the fertilizer disc being 120 r·min^-1^, the calculated value of *H* is 3.2 cm, and the angle should be greater than 53.2°. The distribution chamber designed in this study has a relatively large cavity, with an angle range of approximately 40–60°, as shown in **Figure 7B**, which meets the design requirements.

### 2.8 Test methods

To establish the AFCM and OSMM, we selected various fertilizers for the single-factor test in the bench experiment. The primary term coefficients and constant terms in Equation 3 were obtained through least-squares linear fitting. Subsequently, we established the AFCM and verified the predictability of the model, adaptability of different fertilizers, and control performance of the target fertilizer discharge flow rate in the bench test. In the bench experiment, a full-factorial orthogonal test was conducted and used groove wheel and disc speeds as factors to establish a regression model for the groove wheel speed-disc speed-variable coefficient of each row. The optimal disc speed for different groove wheel speeds was derived from the regression model, and the OSMM was established based on the mapping relationship between the two. A full-factorial test was designed and conducted in the field. The significance of each influencing factor was evaluated using field tests. The differences between the field test results and the model were analyzed to verify the validity and accuracy of the model.

## 3 Tests

### 3.1 Bench test setup

**Figure 8:**
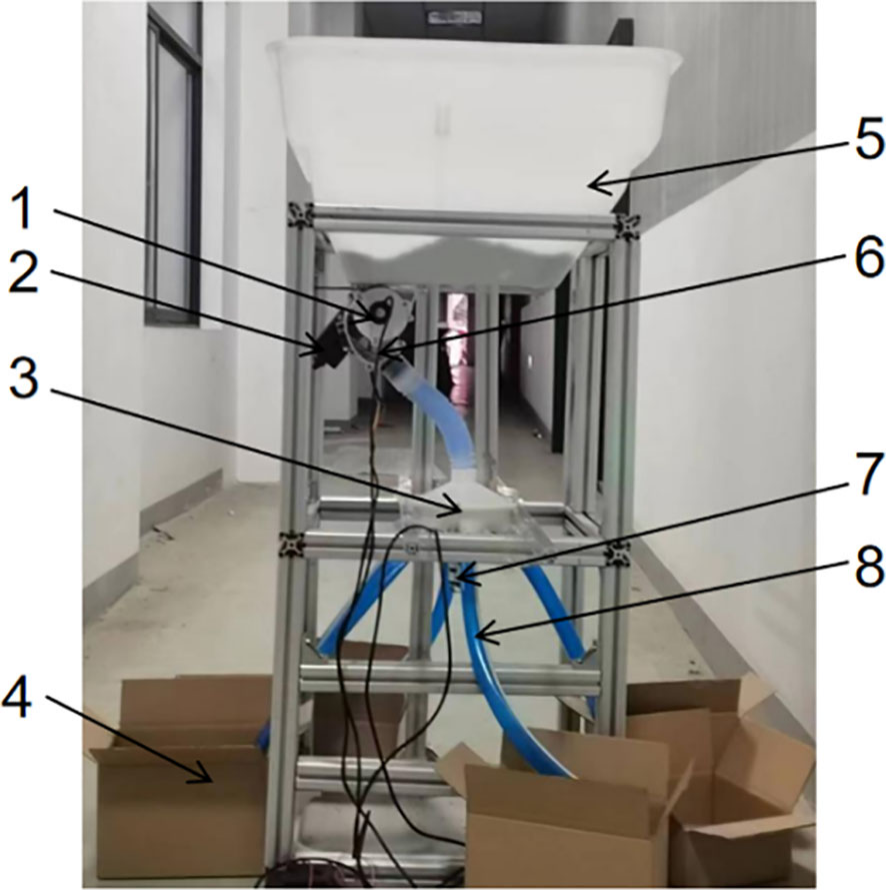
Centrifugal distributor test stand. 1. fertilizer discharger motor encoder 2. fertilizer discharger motor 3. distributor 4. fertilizer receiver box 5 fertilizer box 6. external grooved wheel fertilizer discharger 7. distributor motor 8. fertilizer delivery tube.

The homemade centrifugal distributor test rig used in this study is illustrated in **Figure 8**. The centrifugal dispenser test stand used a fertilizer tank and matching external grooved wheel fertilizer discharger from North Harvest Electronics, which was powered by a matching DC motor. The centrifugal dispenser was a 3D printed part, utilizing PLA as the printing material, powered by a 775 DC gear motor. The test site was located in the Agricultural Machinery Test Center at the College of Engineering, Nanjing Agricultural University, China.

### 3.2 Complete test

#### 3.2.1 Side-depth fertilizer applicator: complete machine

The purpose of the test was to conduct a comprehensive ground test of the overall fertilizer application device to determine its compliance with design requirements. In addition, we analyzed and summarized fertilizer application performance across various working parameters. The overall fertilizer application device was tested for field operation performance to determine whether the performance indices satisfied operational requirements under conditions of vibration and tilting during actual operation.

**Figure 9:**
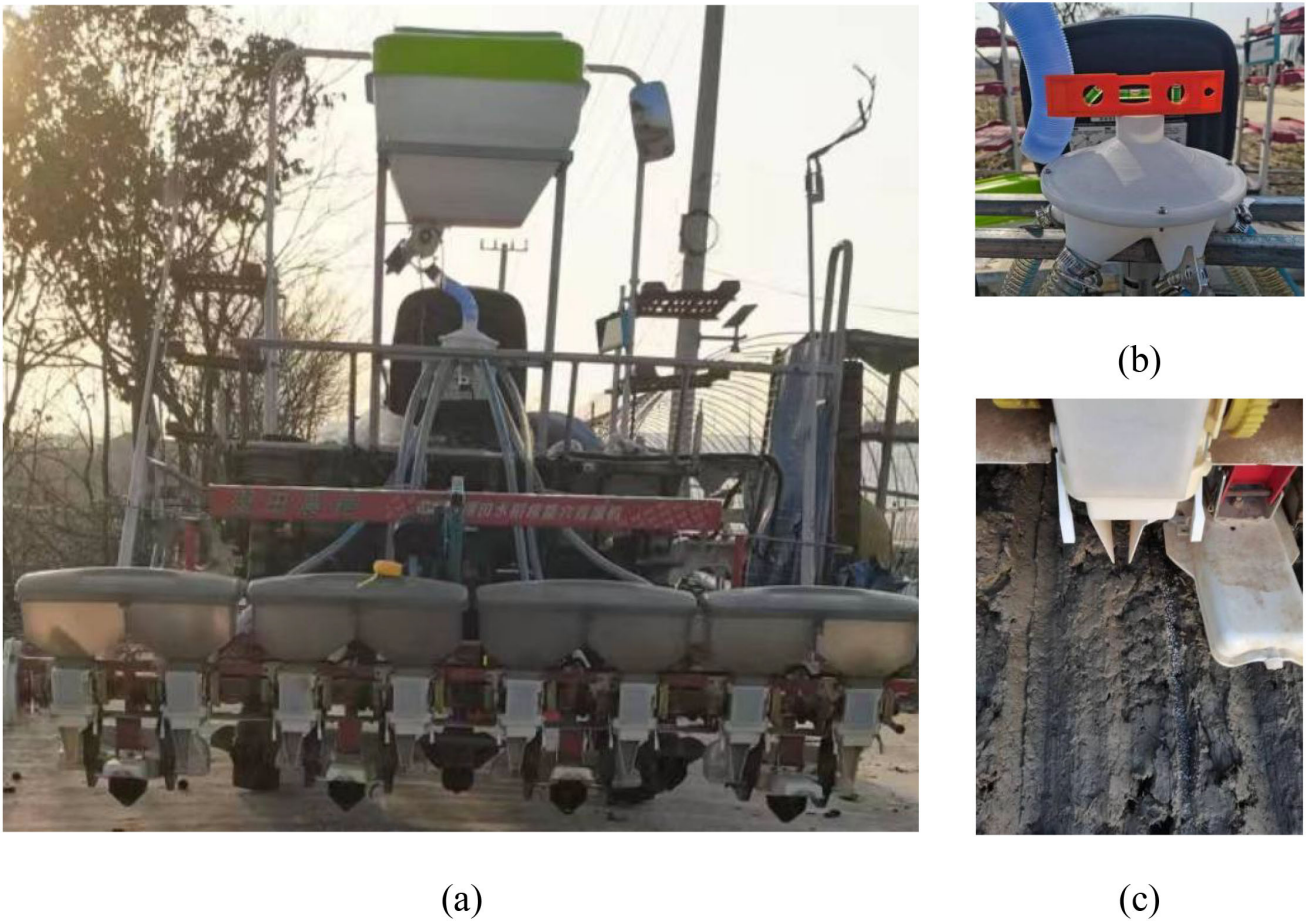
Yanmar YR60D with Side Depth Fertilizer Applicator. **(A)** Side Depth Fertilizer Applicator; **(B)** Distributor Mounting Levels; **(C)** Fertilizer distribution in the field.

The power chassis was a Yanmar YR60D, which featured an adaptive centrifugal distribution fertilizer application device, as shown in **Figure 9**. The field trial was conducted in the Zhuyu Sanhe Field Complex test field in Luhe District, Nanjing.

#### 3.2.2 Experimental design

The overall machine test served three primary purposes: to test and verify the field operation effect of the centrifugal distribution fertilizer applicator; to observe the adaptability of the centrifugal fertilizer discharge system to different fertilizers; and to compare and verify the effectiveness of the control system (refer to the GB/T 20346.1–2006 Test Methods for Fertilizer Applicators). The entire machine test encompassed both dynamic and static evaluations. The mu sowing amount and fertilizer type were selected as test factors. The mu sowing amount ranged from 20 to 50 kg, with four levels chosen. Each operational run covers 100 m, with an operational width of 2.1 m. Consequently, the total target fertilizer application amounts of the four levels were 6,200 g, 9,300 g, 12,400 g, and 15,500 g when a large amount of mu was sown. The speed of the rice transplanter was reduced to achieve the desired total amount of fertilizer application. The target fertilizer discharge rate in the static test was the same as that in the dynamic test. Each group of tests was conducted in triplicate. The actual total amount of fertilizer discharged from the six fertilizer discharge tubes was measured, and the error rate of fertilizer discharge and coefficient of variation between rows were calculated. Simultaneously, the vehicle speed in the dynamic test was monitored using the control system, while the average value of the vehicle speed was recorded. The vehicle speed was used to correlate the experimental results. The experimental design is presented in **Table 1**.

**Table 1:** Centrifugal fertilization experimental design.

Levels	Factors		
	Total target fertilizer application (g)	Fertilizer type	Static or dynamic testing
1	6,200	Slow-release fertilizer	Static
2	9,300	Compound fertilizer	Dynamic
3	12,400	Urea	
4	15,500		

**Figure 10:**
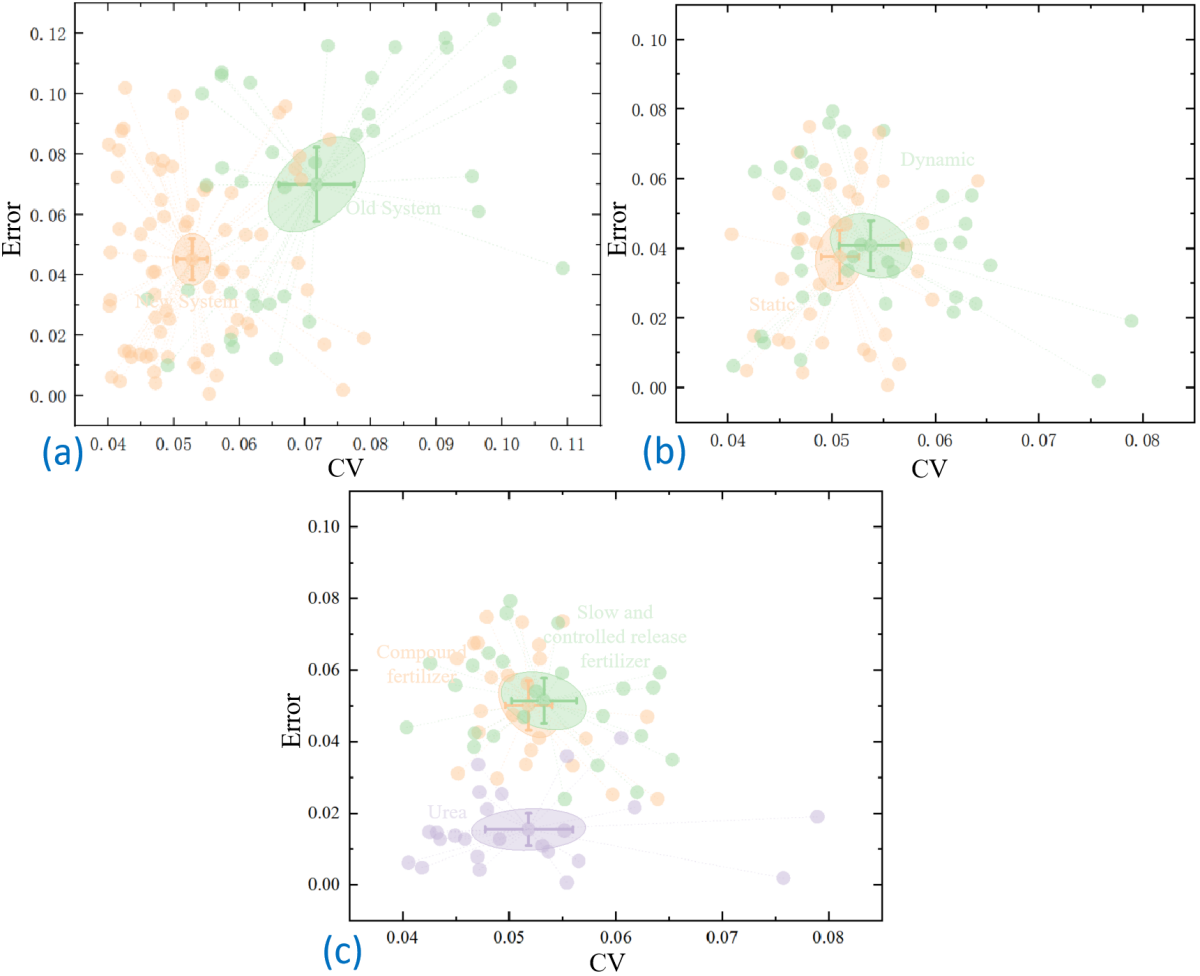
Comparative analysis based on scatterplot **(A)** Comparison of old and new systems **(B)** Comparison of dynamic and static tests **(C)** Comparative analysis of different fertilizers.

In addition, a traditional control system was employed to conduct a static test on the target discharge of fertilizer at four levels: 6,200 g, 9,300 g, 12,400 g, and 15,500 g. The test materials were a slow-mixed fertilizer, compound fertilizer, and three kinds of urea fertilizer. The control model of the experimental fertilizer discharge was selected from the traditional calibration formula. The speed of the distributor was set to 100 rpm. The PID control parameters of the distributor and fertilizer discharger drive motors were kept the same. The experimental design is presented in **Table 2**. The experimental results are not shown in the **Table 2** but only in the results analyzed in **Figure 10**.

**Table 2:** Static test design without a control system.

Levels	Factors	
	Total target fertilizer application (g)	Fertilizer type
1	6,200	Slow-release fertilizer
2	9,300	Compound fertilizer
3	12,400	Urea
4	15,500	

### 3.3 Test materials

The test materials encompassed cardboard boxes, handheld electronic scales, stopwatches, tape measures, and data-recording forms. Three fertilizer types were selected: slow-release fertilizer, urea, and compound fertilizer. The specific brands and physical properties of the fertilizer types are shown in **Table 3**.

**Table 3:** Fertilizer information sheet.

Fertilizer type	Slow-release fertilizers	Compound fertilizer	Urea
Brand	Hanfeng Evergreen Inc	Stanley Agricultural Group Co., Ltd.	Dongpinghu Brand
Bulk density (g/cm^3^)	0.82	0.90	0.92
	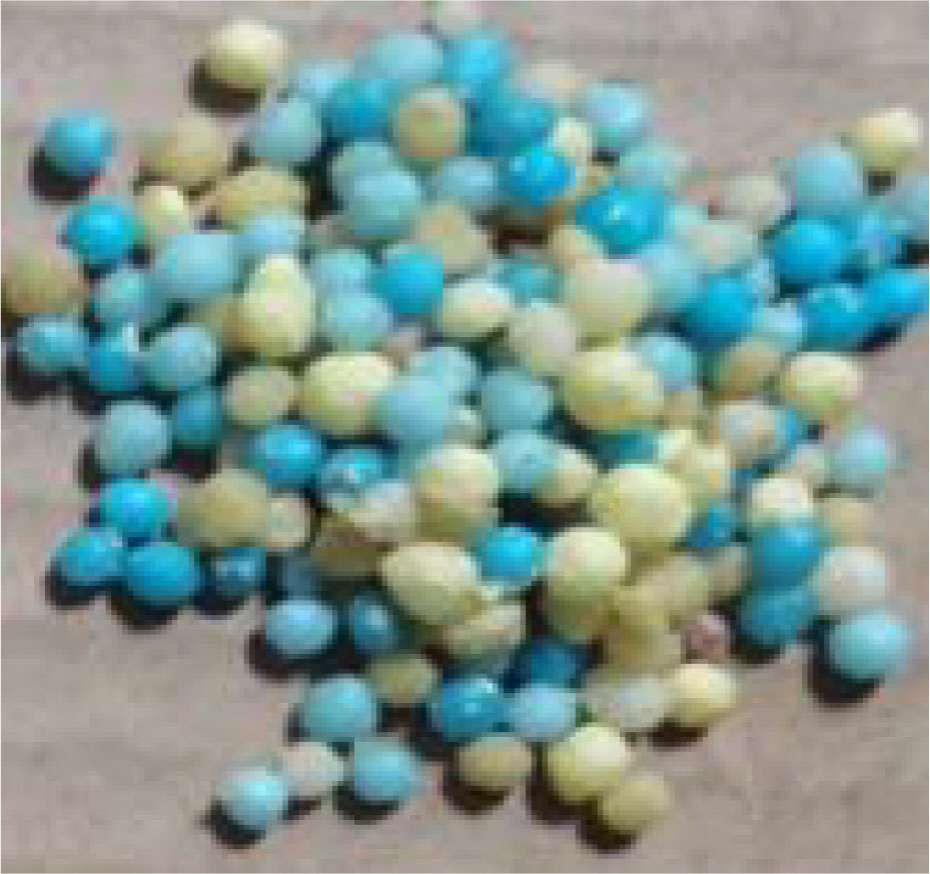	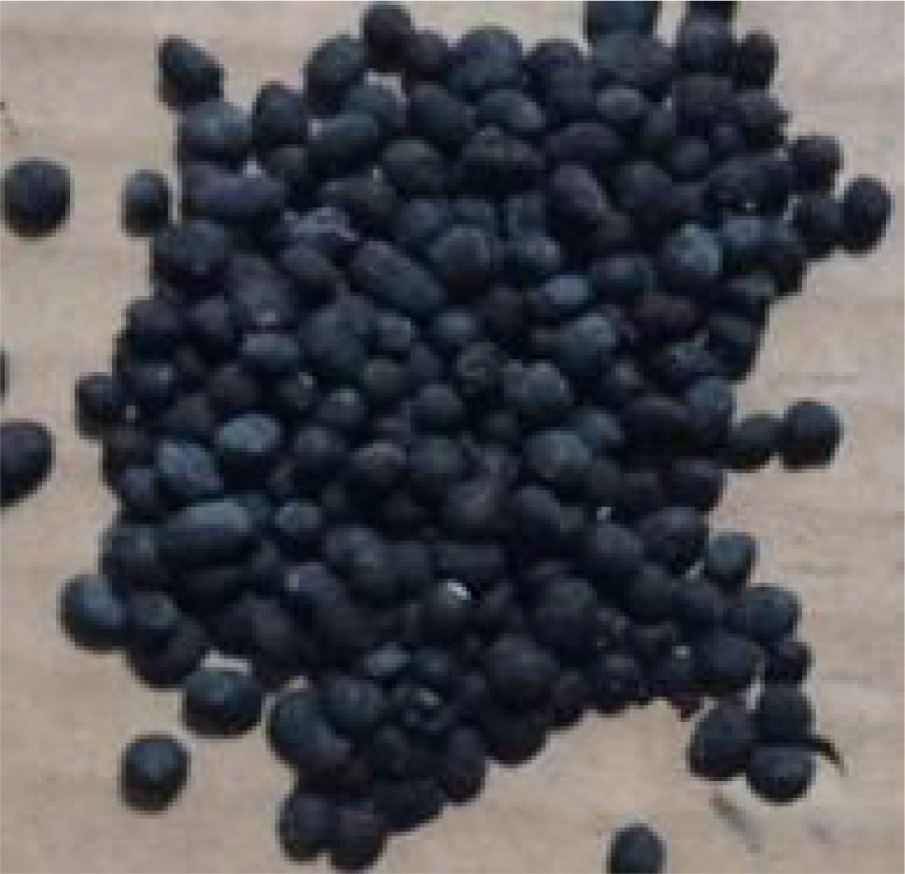	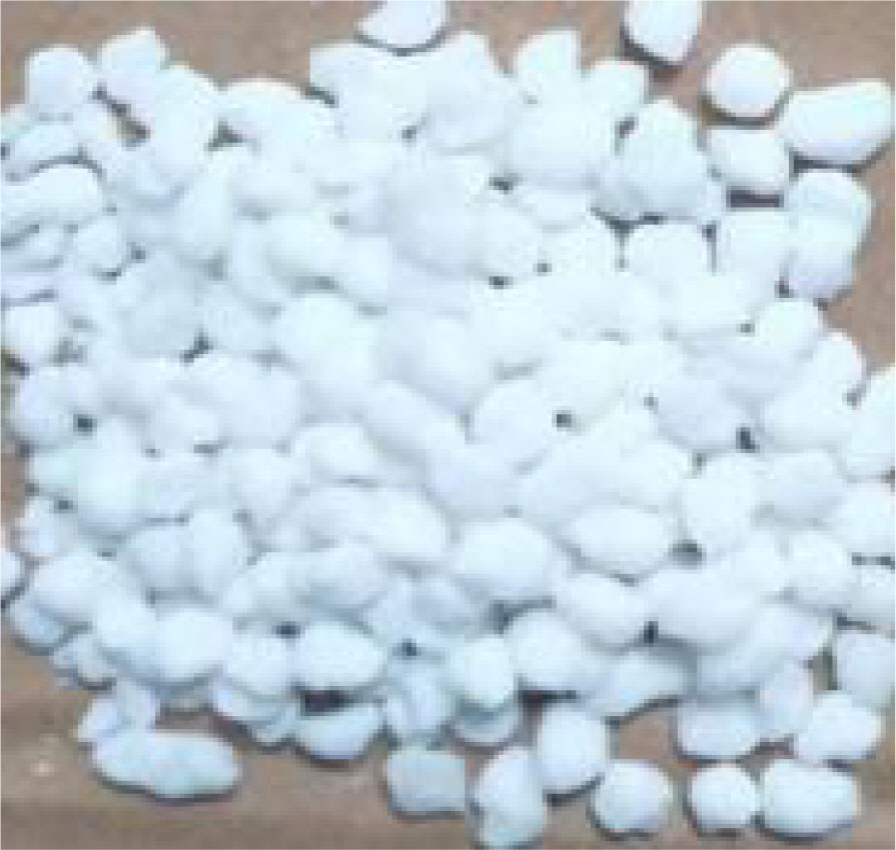

### 3.4 Test indicators

(1) Coefficient of variation for consistency of fertilizer discharge across rows.

The experiment involved the selection of six fertilizer discharges, which were performed in triplicate and averaged. The coefficient of variation for consistency of fertilizer discharge in each row was calculated using to (Equations 23–25).

(23) $$a_{h}=\frac{\sum_{x=1}^{n_{h}}a_{x}}{n_{h}}$$

(24) $$S_{h}=\frac{\sqrt{\sum_{x=1}^{n_{h}}(a_{x}-a_{h}{)}^{2}}}{n_{h}-1}$$

(25) $$CV=\frac{S_{h}}{a_{h}}\times 100\%$$

where *a_h_* is the mean value of fertilizer discharge in each row, g; *a_x_* is the amount of fertilizer discharged in the *xth* row, g; *S_h_* is the standard deviation of fertilizer discharge in each row; CV is the coefficient of variation of the consistency of discharges in each row; and $n_{h}$ is the number of rows to be measured.

(2) Error in the total amount of fertilizer discharged.

Various target fertilizer discharge volumes were set, the fertilizer discharged from each discharge port was performed in triplicate, and the total error in fertilizer discharge was calculated according to (Equation 26). The average error in fertilizer discharge in multiple trials was calculated using (Equation 27):

(26) $$\delta_{m}=\frac{M_{s}-M_{m}}{M_{m}}\times 100\%$$

(27) $$MAE=\frac{1}{n}\sum_{i=1}^{n}\delta_{m}$$

where, $\delta_{m}$ is the discharge error, %; *M_S_* is the actual fertilizer volume value, kg; *M_m_* is the target value, kg; *MAE* is the mean absolute error, %.

## 4 Results and discussion

### 4.1 Calibration test results

#### 4.1.1 Adaptive flow control modeling

The rotational speed of the fertilizer discharge shaft was selected as 20–80 rpm, with increments of 10 rpm, resulting in seven distinct levels. Urea, compound fertilizer, and slow-mixed fertilizer were selected as the test objects, with a 1-min fertilizer discharge time. The test results are presented in **Table 4** (each test group was performed in triplicate to obtain the average value).

**Table 4:** One-minute displacements of various fertilizers at different speeds.

Fertilizer type		Speed (rpm)						
		20	30	40	50	60	70	80
Displacement (g)	Urea	3,505.00	5,192.50	6,860.00	8,458.33	10,025	11450.83	12,900
	Slow-release fertilizer	2,906.67	4,337.50	5,766.67	7,179.17	8,540	9846.67	11,246.67
	Compound fertilizer	3,346.67	4,977.5	6,593.33	8,187.5	9,765	11,287.5	12,826.67

**Figure 11:**
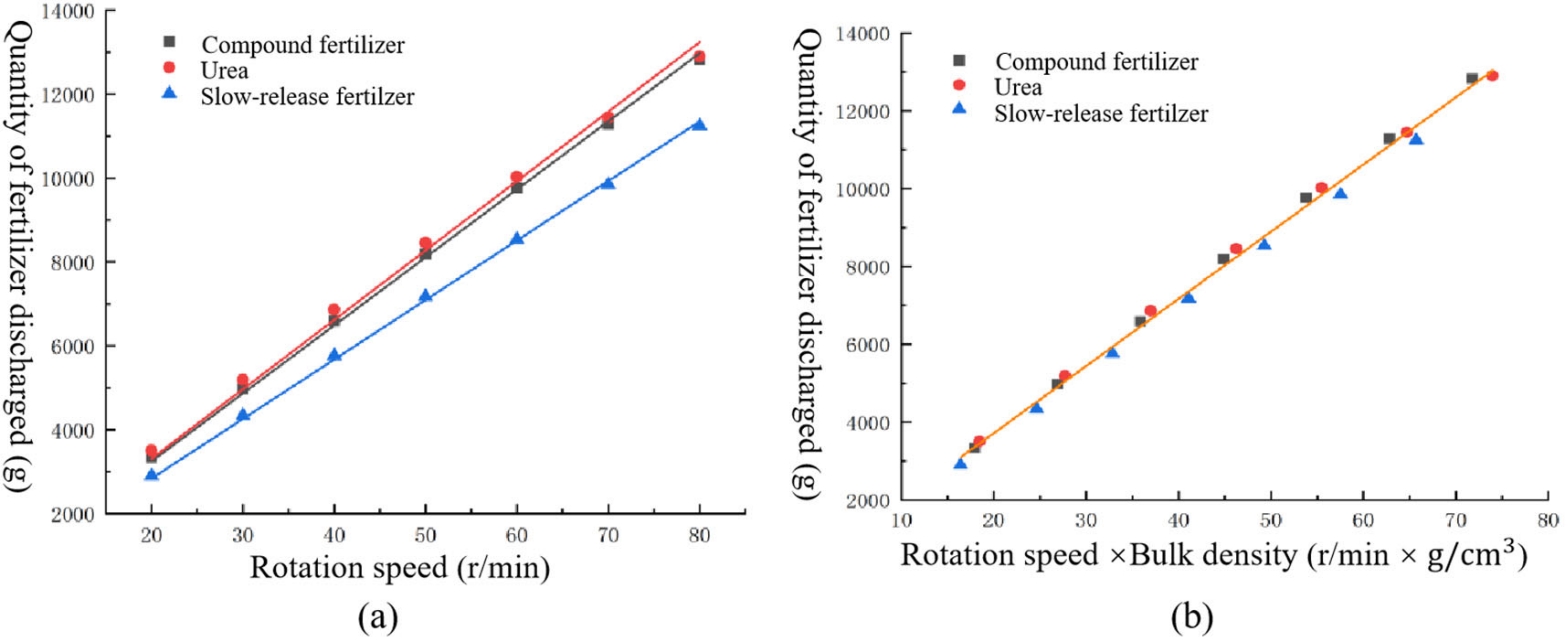
Comparison of different fitting methods. **(A)** Conventional formula fitting. **(B)** Introduction of bulk density fitting.

The data in **Table 4** were fitted as shown in **Figure 11** using the conventional calibration equation (Equation 2) and the new calibration equation (Equation 3), respectively:

**Figure 11A** illustrates that the conventional calibration formula (Equation 2) produces distinct fitting curves for compound fertilizers, urea, and slow-mixed fertilizers because of their different bulk densities. The conventional calibration formula considers bulk density as an intrinsic attribute and disregards its impact. This results in the use of the conventional calibration formula for the calibration of the application of the fertilizer control system. Therefore, it can only be used to accurately control the amount of discharge for a type of fertilizer. Switching the fertilizer type complicates the precise control of discharge.

**Figure 11B** illustrates that the new calibration equation (Equation 3) demonstrates a linear relationship between the displacements of the three fertilizers, taking into consideration the effect of bulk density. The fitting model is represented by (Equation 28), with a coefficient of determination of R^2^ = 0.996.

(28) $$Q=172.6\rho^{\prime }n+261.91$$

where *Q* is the flow rate of the fertilizer discharge, g/min; $\rho^{\prime }$ is the bulk density of the fertilizer, g/cm^3^; *n* is the rotational speed of the fertilizer discharge shaft, rpm.

#### 4.1.2 Adaptive flow control system validation

For fertilizer applicators, according to (Equation 29), changing the mu of fertilizer and the forward speed of the machine is equivalent to changing the fertilizer applicator discharge rate. Therefore, the forward speed and mu of the machine can be replaced by the rate of fertilizer discharge. The fertilizer discharge rate and fertilizer type were selected as the test factors. The operating speed of common market paddy field operation machinery was 3–5 km/h. The width was 2.1–2.2 m, the fertilizer application amount was 200–600 kg/ha, and the required fertilizer discharge rate could be calculated as 2.1–11.0 kg/min. In terms of the discharge volume of the fertilizer applicator selected in this experiment, the fertilizer discharge rate was selected as 2,000–12,000 g/min, with increments of 2,000 g/min. and six levels. With a fertilizer discharge time of 1 min, the fertilizer was still used in the above-mentioned three fertilizer types, and each group of tests was conducted in triplicate to obtain the average values. Three levels were used to investigate the adaptability of the control system to uncalibrated fertilizers.

**Figure 12:**
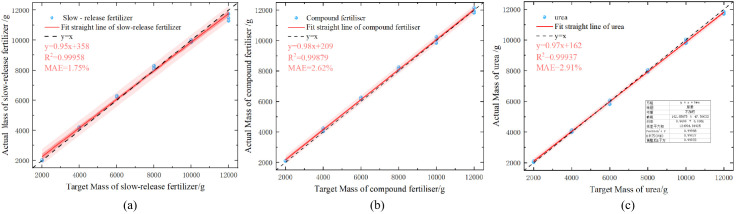
Fertilizer discharge test results based on a novel calibration formula. **(A)** Slow-release fertilizer **(B)** Compound fertilizer **(C)** Urea.

(29) Q=25qvb

where *Q* is the flow rate of the fertilizer discharge, g/min; *q* is the amount of mu, kg/mu; *v* is the forward speed of the fertilizer applicator, km/h; *b* is the operational width, m. As shown in **Figure 12**, the $R^{2}$ of the linear fit of the three fertilizers were all greater than 0.99, thereby indicating that the linearity of the fertilizer discharge device was superior, and a consistent discharge volume could be maintained at different discharge rates. The average absolute errors for slow-release fertilizers, compound fertilizers, and urea were 1.75%, 1.62%, and 2.91%, respectively. Comparing the calculated values obtained from the model with the available experimental data, the maximum error in the discharge of slow-mixed fertilizer at seven speeds $\delta_{m}$ was 6.58%; the maximum error in the discharge of compound fertilizer at seven speeds $\delta_{m}$ was 2.2%; and the maximum error in the discharge of urea at seven speeds $\delta_{m}$ was 3.12%.

The bench test demonstrated that the adaptive fertilizer application control system designed in this study exhibited strong adaptability and high accuracy for the three fertilizer types.

#### 4.1.3 Comparison tests with the original model

**Figure 13:**
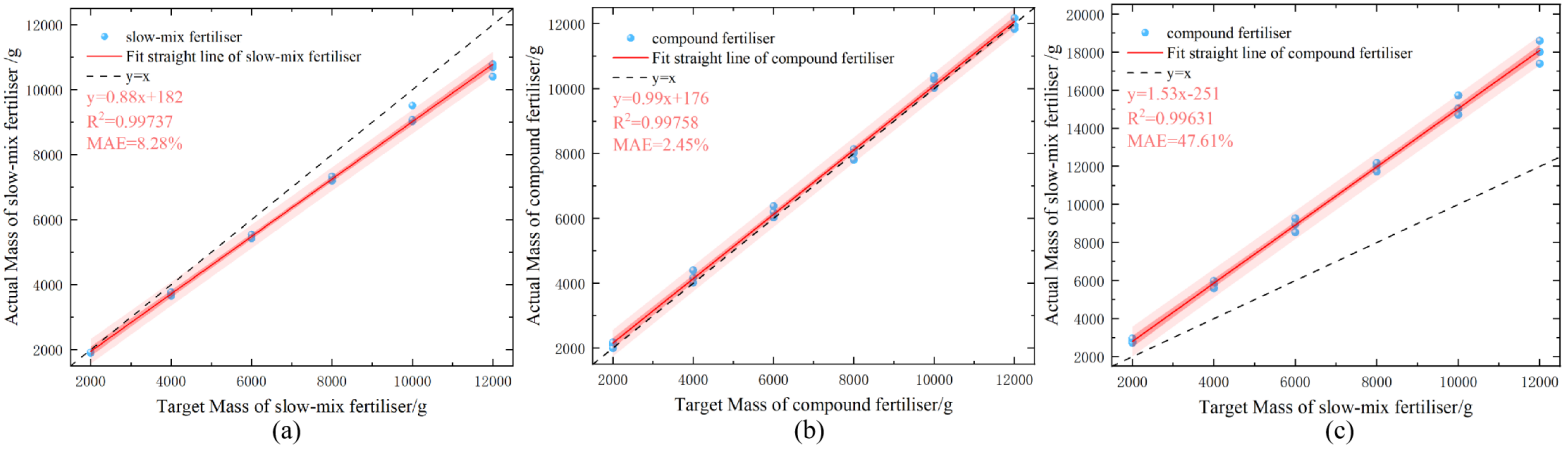
Fertilizer discharge test results for conventional calibration model. **(A)** Slow-release fertilizer **(B)** Compound fertilizer **(C)** Urea.

The conventional control system was calibrated using a compound fertilizer. The same procedure was performed using slow-mixed fertilizer, compound fertilizer, and urea, and the data were recorded. The test results are presented in **Figure 13**. The results showed that the average errors of the control system using the conventional calibration formula with slow-mixed fertilizer, compound fertilizer, and urea were 8.28%, 2.45%, and 47.6%, respectively.

In the fertilizer discharge tests of slow-release mixed fertilizer and urea, the error of the traditional model was 6.53% and 44.7% higher than that of the new model, respectively, with both showing a significant increase. The measurements indicated that an increased disparity between the bulk density of the fertilizer and calibration benchmark correlated with a more significant error. By dynamically introducing the bulk density parameter, the new formula reduced the error rate of different fertilizer discharges to within 2.91%.

### 4.2 Optimal speed matching modeling and experimental validation

A response surface test was conducted to further investigate the distribution effect of the centrifugal distributor on the external tank wheel-type fertilizer discharger. This test examined the interaction between various parameters of the distributor at different rotational speeds of the external tank wheel-type fertilizer discharger. The rotational speeds of the external tank wheel fertilizer discharger and distributor were selected as the influencing factors, while the CV was used as the response value. The rotational speed of the fertilizer discharger was selected as 20–80 rpm, while that of the distributor was 100–140 rpm. The experimental design was performed using Design-Expert 12 software, and the results of the Central Composite response surface method test are listed in **Table 5**.

**Table 5:** Central composite response surface method test results.

Serial number	Considerations		CV (%)
	Rotating speed of fertilizer discharger (RPM)	Dispenser speed (RPM)	
1	80	100	4.30
2	50	91.7157	3.34
3	50	120	2.91
4	7.57359	120	2.68
5	50	120	3.11
6	80	140	2.67
7	20	100	2.69
8	92.4264	120	3.72
9	50	148.284	2.77
10	50	120	2.92
11	20	140	2.85
12	50	120	2.83
13	50	120	3.04

From the test results, the CV for row-wise consistency varied between 2.67% and 3.72%. The ANOVA results of the response surface tests using the CCD-RSM method are listed in **Table 6**. The F-value of the regression equation was 34.68, the P-value was<0.001, and the model achieved a highly significant level. This indicates that the model has good fitting accuracy and can be used to conduct subsequent design optimization using the response surface approximation model. The P-value of the deformation term was 0.3476 (P > 0.05), indicating that the deformation term was not significant and that the model had a good degree of fit. From the size of the F-value of each factor in the table, it can be concluded that the effect of the fertilizer discharger rotational speed on the CV was higher than that of the distributor rotational speed. The R^2^ of the regression model was 0.9612, and the difference between the predicted R^2^ (0.8263) and adjusted R^2^ (0.9335) was less than 0.2, which indicated that the model was well-fitted; the measured signal-to-noise ratio was 19.4554 > 4, indicating that the signals were adequate; and the CV value was 4.00%< 10%, indicating that the test had high confidence and accuracy.

**Table 6:** ANOVA results.

Source	Square sum	df	Mean square	F-value	P-value
model A	2.61	5	0.5211	34.68	< 0.0001
	1.05	1	1.05	70	< 0.0001
B	0.6476	1	0.6476	43.1	0.0003
C	0.801	1	0.801	53.31	0.0002
AB	2.61	5	0.5211	34.68	< 0.0001
A²	0.0985	1	0.0985	6.56	0.0375
B²	0.015	1	0.015	1	0.3504
Residual	0.1052	7	0.015		
Lack of Fit	0.0553	3	0.0184	1.48	0.3476
Pure error	0.0499	4	0.0125		
Cor Total	2.71	12			

**Figure 14:**
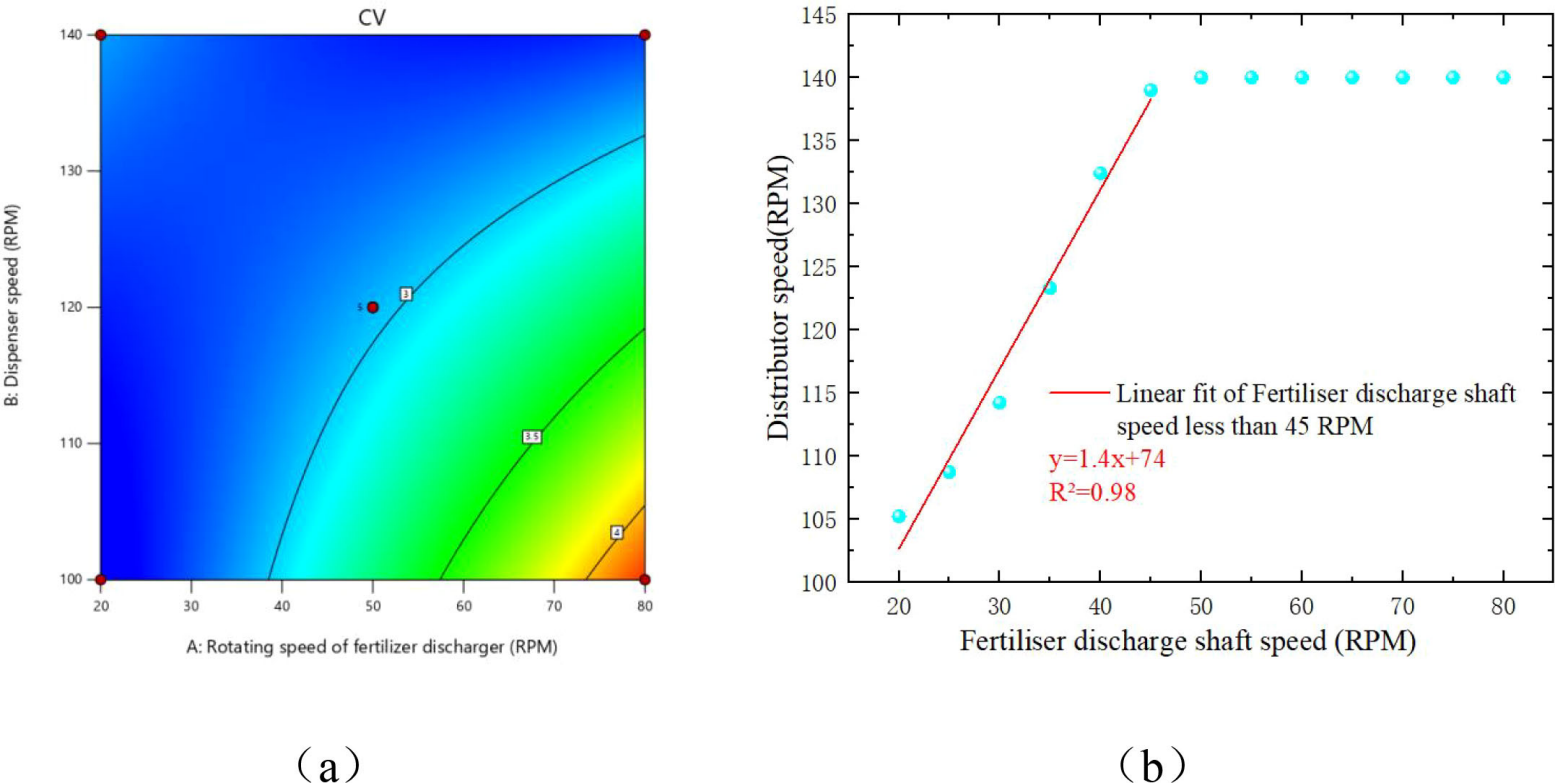
Optimal distributor speed model. **(A)** Contour map of the optimal distributor speed matching model. **(B)** Two-dimensional form of the optimal distributor speed model.

Derive the fitted response surface equation as.

(30) $$y=2.96+0.3626A-0.2845B-0.4475AB+0.1190A^{2}+0.0465B^{2}$$

(Equation 30) is the three-dimensional form of the OSMM, and its response surface representation is shown in **Figure 14A**. The optimal distributor speed was solved for different fertilizer discharge shaft speeds by setting the boundary conditions, as shown in (Equation 31).

(31) $$\{\begin{array}{c} \min CV\\ A\in Terget\;Value\\ B\in \left[100,140\right] \end{array}$$

However, the response time of running this 3D model on the STM32 microcontroller was slow; therefore, in this study, we found the optimal B under different A with the goal of minimizing the CV value in Design Expert 13.0 by plotting the scatter plot, as shown in **Figure 14B**. We divided the scatter plot into two segments to obtain an OSMM with a segmented function, as illustrated in (Equation 32).

(32) $$\{\begin{array}{c} \begin{array}{cc} B=1.4A+74 & \;\;0<A\leq 45 \end{array}\\ \begin{array}{cc} B=140 & \;45<A<80 \end{array} \end{array}$$

### 4.3 Complete machine test results

#### 4.3.1 Test results

The purpose of the overall machine test was to verify the performance of the centrifugal distribution fertilizer application system mounted on the machine. The mounted control system was tested as a full-factorial test with the target fertilizer discharge rate and fertilizer type. Static and dynamic tests were performed, with the results presented in **Table 7**.

**Table 7:** Field dynamic test results.

Num	Target Mass (g)	Fertilizer Type	Dynamic or Static	CV (%)	Average Vehicle Speed (m/s)	MAE (%)	Average Fertilizer Discharge(g)
1	6,200	1	1	5.97	0	2.52	6,356.45
2	9,300	1	1	4.89	0	4.97	9,761.97
3	12,400	1	1	4.49	0	7.86	13,374.51
4	15,500	1	1	5.17	0	7.62	16,681.53
5	6,200	2	1	6.91	0	7.93	5,708.29
6	9,300	2	1	5.75	0	7.41	8,611.19
7	12,400	2	1	5.46	0	6.81	11,555.99
8	15,500	2	1	6.33	0	3.88	14,897.89
9	6,200	3	1	5.31	0	1.08	6,267.25
10	9,300	3	1	4.25	0	1.48	9,437.37
11	12,400	3	1	4.72	0	0.42	12,452.11
12	15,500	3	1	5.65	0	0.67	15,396.73
13	6,200	1	2	6.89	0.82	4.40	6,472.80
14	9,300	1	2	4.83	0.57	7.78	8,576.91
15	12,400	1	2	5.12	0.41	7.74	13,359.40
16	15,500	1	2	5.88	0.33	2.10	15,174.50
17	6,200	2	2	7.03	0.79	3.50	6,417.00
18	9,300	2	2	5.01	0.66	6.71	8,675.81
19	12,400	2	2	4.67	0.42	7.86	13,374.43
20	15,500	2	2	6.12	0.38	2.40	15,128.00
21	6,200	3	2	6.05	0.87	4.10	6,454.20
22	9,300	3	2	4.93	0.62	7.91	8,564.26
23	12,400	3	2	4.32	0.44	5.49	13,081.10
24	15,500	3	2	5.54	0.32	3.60	16,058.00

The average value of MAE of fertilizer discharged in the overall machine test was 4.84%, while the maximum MAE was 7.93%. This satisfied the operational requirements. The average value of the CV of fertilizer discharge in each row was 5.47%, while the maximum CV was 7.03%. This satisfied the operational requirements.

#### 4.3.2 Correlation analysis

**Figure 15:**
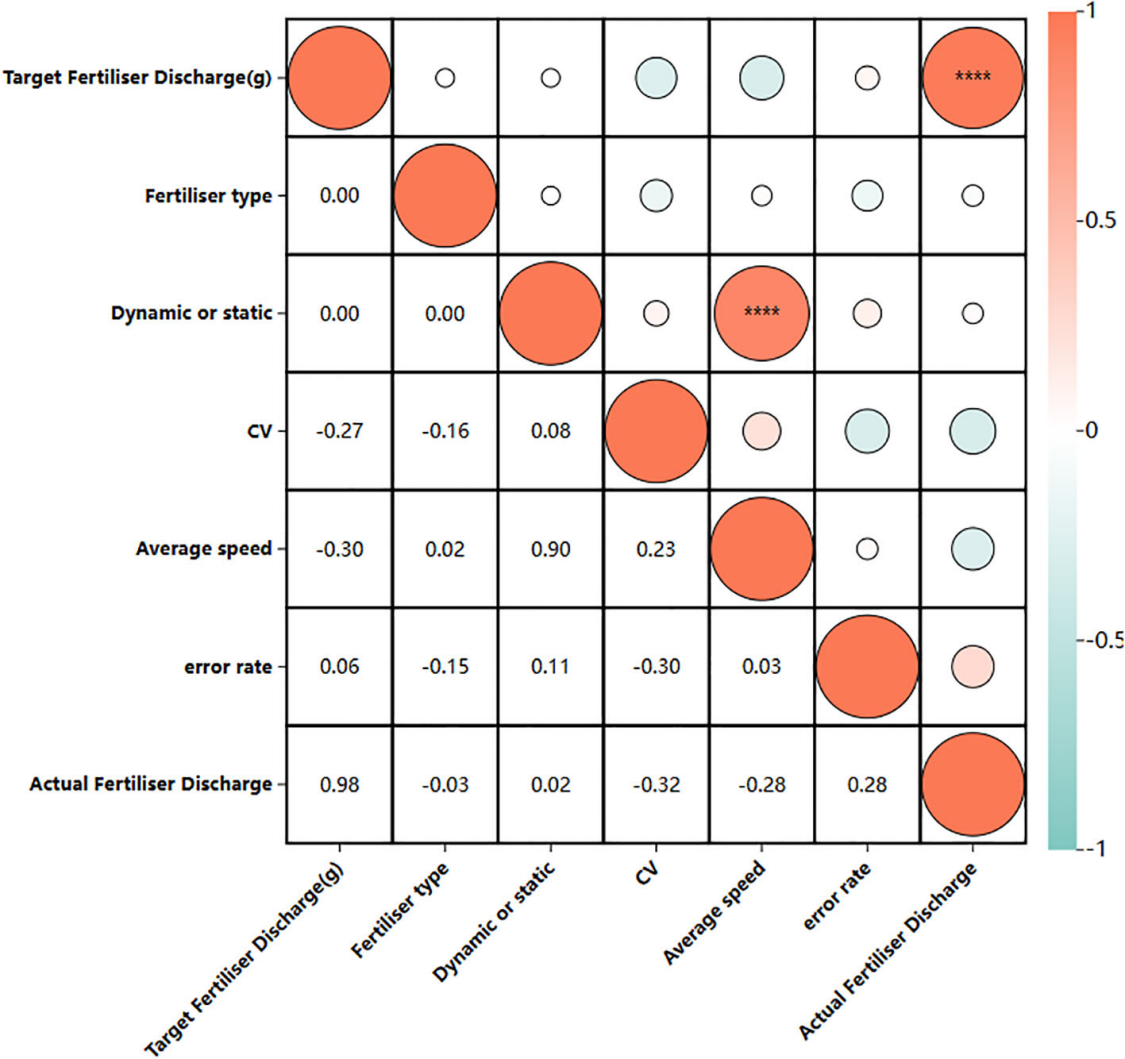
Pearson correlation analysis.

**Figure 15** illustrates the verification of the anti-interference capabilities of the new fertilizer discharge control system via Pearson correlation analysis. The analysis utilized data from the dynamic/static test (static code “1” speed is 0, dynamic code “2”). The fertilizer types examined included slow-mixed fertilizer, compound fertilizer, and urea, which were categorized according to their bulk density in ascending order (coded as 1/2/3). The results showed that the target fertilizer discharge and the actual amount of fertilizer discharged were strongly positively correlated (r > 0.8). Additionally, test-type coding was significantly correlated with vehicle speed, which aligned with system design expectations. The correlation coefficients between fertilizer type and discharge error rate and row-by-row CV were -0.15 and -0.16, respectively (absolute value<0.3 threshold), thereby confirming that differences in the physical properties of fertilizers (e.g., urea bulk density is 18.4% lower than compound fertilizer) had a weak effect on accuracy. The correlation coefficients between dynamic/static conditions and discharge error and CV were only 0.11 and 0.08, indicating that vibration and other disturbing factors were suppressed and the system was resistant to environmental impact. The correlation coefficients of dynamic/static working conditions and fertilizer discharge error and CV were merely 0.11 and 0.08, indicating that field vibration and other disturbing factors were suppressed.

#### 4.3.3 Comparative analysis

This section seeks to analyze the differences between the errors in the amount of fertilizer discharged and the coefficients of variation across rows for old and new systems, operating conditions, and fertilizers using visual scatter plots.

**Figure 10A** illustrates that the average values of the CV of each row and the total fertilizer discharge error in the static test of the new system are 0.049 and 0.051, respectively. Conversely, the average values of the CV of each row and the total fertilizer discharge error for the traditional system were 0.072 and 0.060, respectively. Consequently, the new system demonstrated improvements of 31.9% and 15.0% in the CV for each row and the total fertilizer discharge error, respectively, when compared to the traditional system.

**Figure 10B** illustrates that the average CV values of each row and the total fertilizer discharge error were 0.055 and 0.052, respectively, in the dynamic test with the control system, while in the static test, they were 0.050 and 0.053. Consequently, the average CV values for each row and the total fertilizer discharge error increased by -10.0% and 2.0%, respectively, in the dynamic test compared to the static test. However, minimal variation was observed compared to the static test.

**Figure 10C** illustrates that the urea exhibited the lowest discharge error, with a mean value of approximately 0.016. Conversely, the compound and slow-release fertilizers demonstrated higher discharge errors of 0.051 and 0.050, respectively. The coefficients of variation of the three fertilizers did not differ significantly, measuring 0.052, 0.052, and 0.053, respectively.

### 4.4 Discussion

In the experimental design of this study, we first introduced an innovation: adopting the target fertilizer discharge volume as the unified independent variable. On this basis, we established a comparability framework by integrating two types of data: dynamic data (from real-time monitoring of natural vehicle speed) and static data (from target value-time dual control). Specifically, in the dynamic test, the total fertilizer required for a 100-meter application was used as the benchmark for the static test, with the static fertilizer discharge time limited to 30 seconds. During the test, the system’s adaptive mechanism enabled real-time dynamic responses of key parameters, such as groove wheel rotational speed and distributor speed. Compared with traditional methods— which only rely on a single independent variable (e.g., vehicle speed, seeding rate per mu, or groove wheel speed) and thus fail to capture parameter interactions—our design establishes a dynamic response relationship between key parameters (e.g., groove wheel speed and distributor speed). Building upon this experimental framework, the analysis presented in Section 4.3.2 was conducted.

**Table 8** lists the common side-deep fertilizer applicators used in paddy fields, among which the first one is the fertilizer applicator developed in this study. Among them, the mass of the one-row Mechanical Fertilizer Dispenser+ with pneumatic conveying was the largest, up to 77 kg, and the mass of the one-row Mechanical Fertilizer Dispenser+ with pneumatic distribution was the lightest, up to 32 kg. However, the multi-row Mechanical Fertilizer Dispenser in the present study had a mass of only 13 kg. This represents a considerable reduction in the mass of the fertilizer application device, achieving a decrease of 16–40% compared to that of the original model and positioning it at the forefront of the industry. At the same time, the pneumatic conveying fertilizer applicator requires 0.38–1.6 kW power, the pneumatic distribution fertilizer applicator needs 2.35 kW, and the mechanical fertilizer applicator with one vessel and one row requires 0.40–0.50 kW power. Conversely, the fertilizer applicator designed in this study requires only 0.3 kW power. Therefore, it represents the lowest tier in the industry for energy efficiency and can conserve power for side-deep furrowing.

**Table 8:** Quality and power parameters of various paddy fertilizer applicators.

Num	Type	Source	Product picture	Mass (kg)	Power (kW)
1	Mechanical fertilizer drainage + mechanical distribution	The fertilizer applicator designed in this study	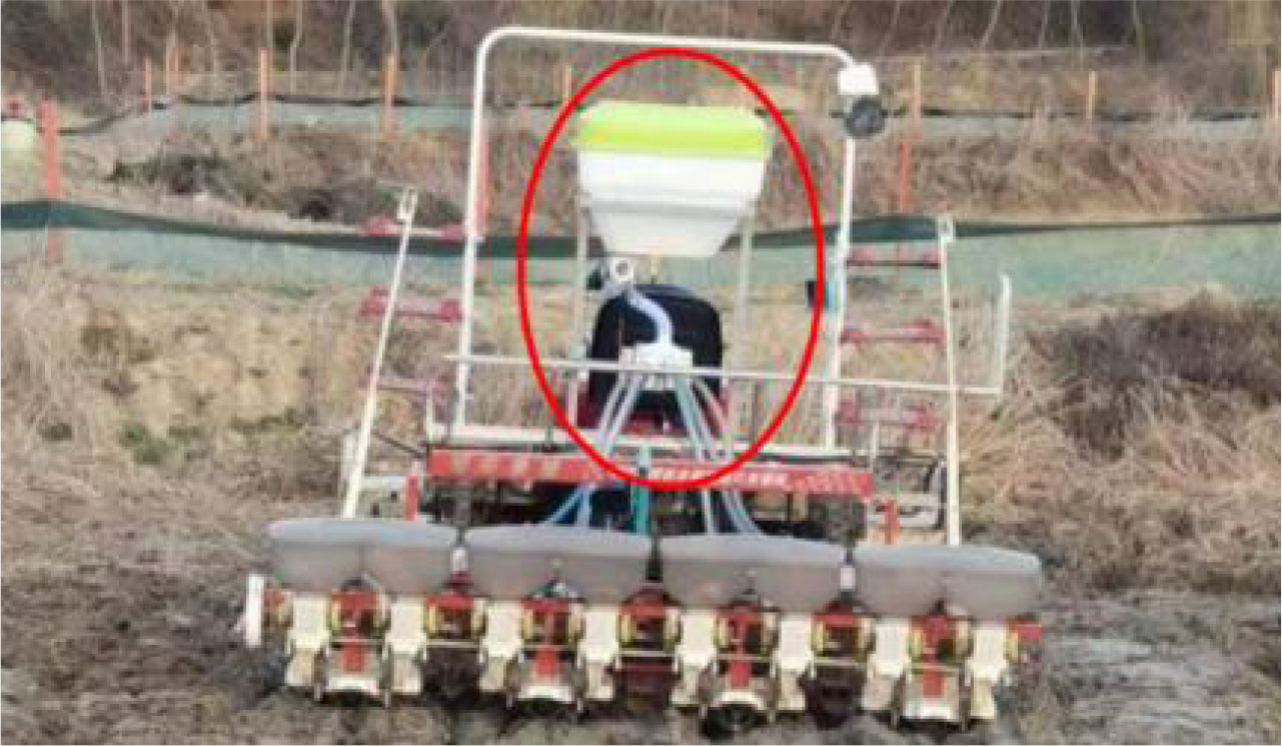	13	0.30
2	Purely mechanical fertilizer application	Hunan Longzhou Agricultural Machinery Co., Ltd.	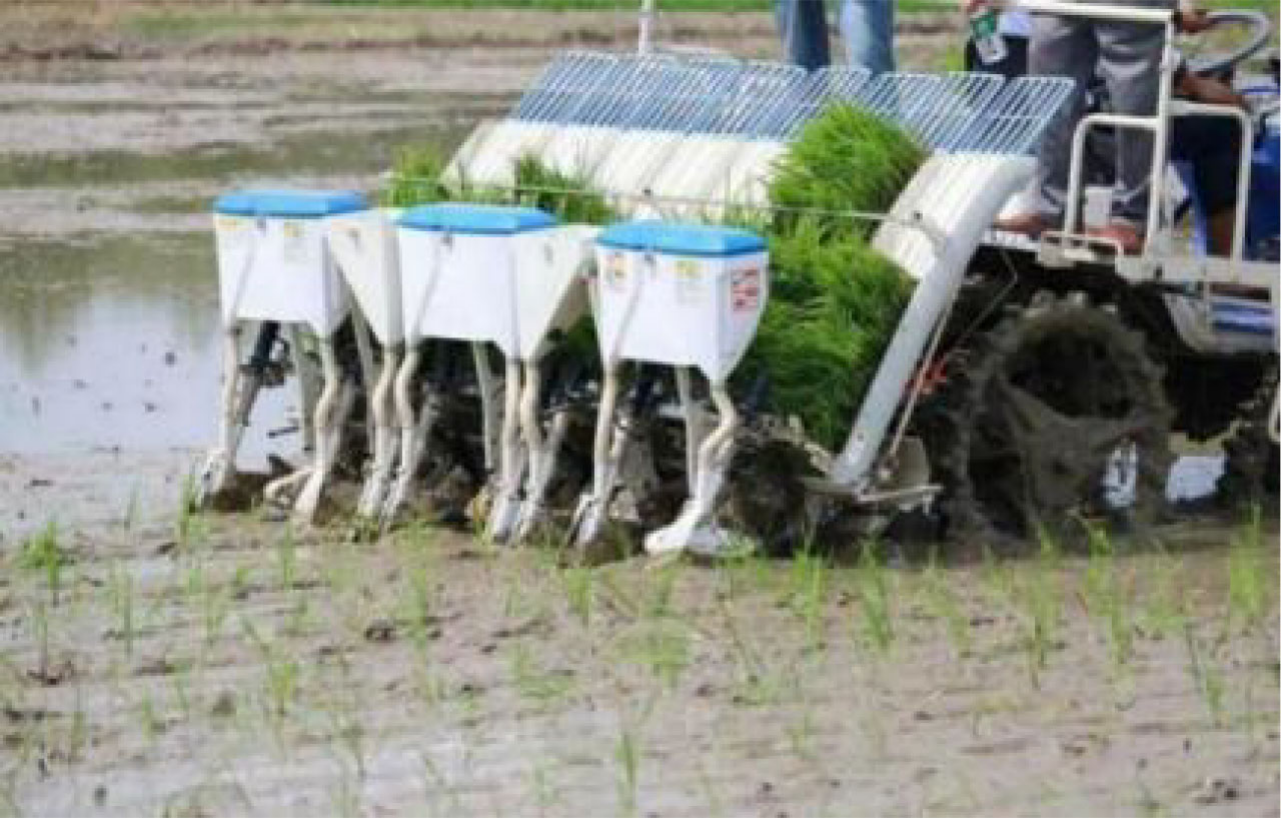 Longzhou 2FH-6 Precision Fertilizer Applicator	48	0.40–0.50
3	Mechanical fertilizer discharge + air-assisted	Yanmar Agricultural Machinery (China) Co., Ltd.	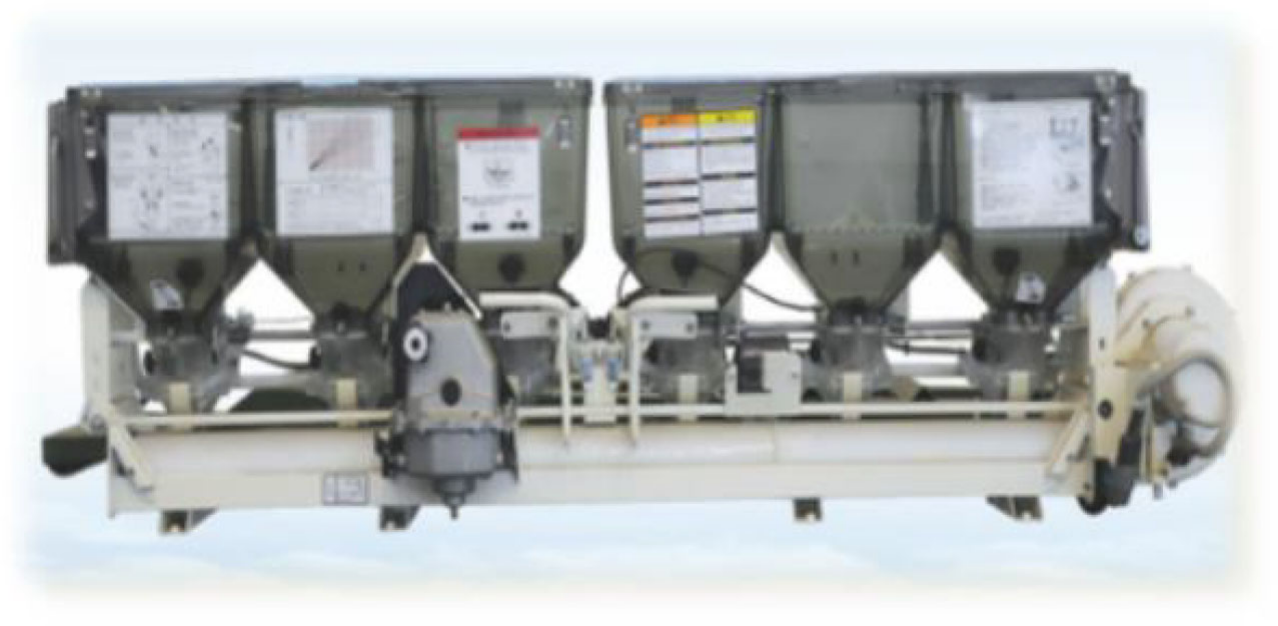 Yanmar 2FC-6 Rice Side Deep Fertilizer Applicator	77	1.30–1.60
4	Mechanical fertilizer discharge + air distribution	(Zhang, 2022)	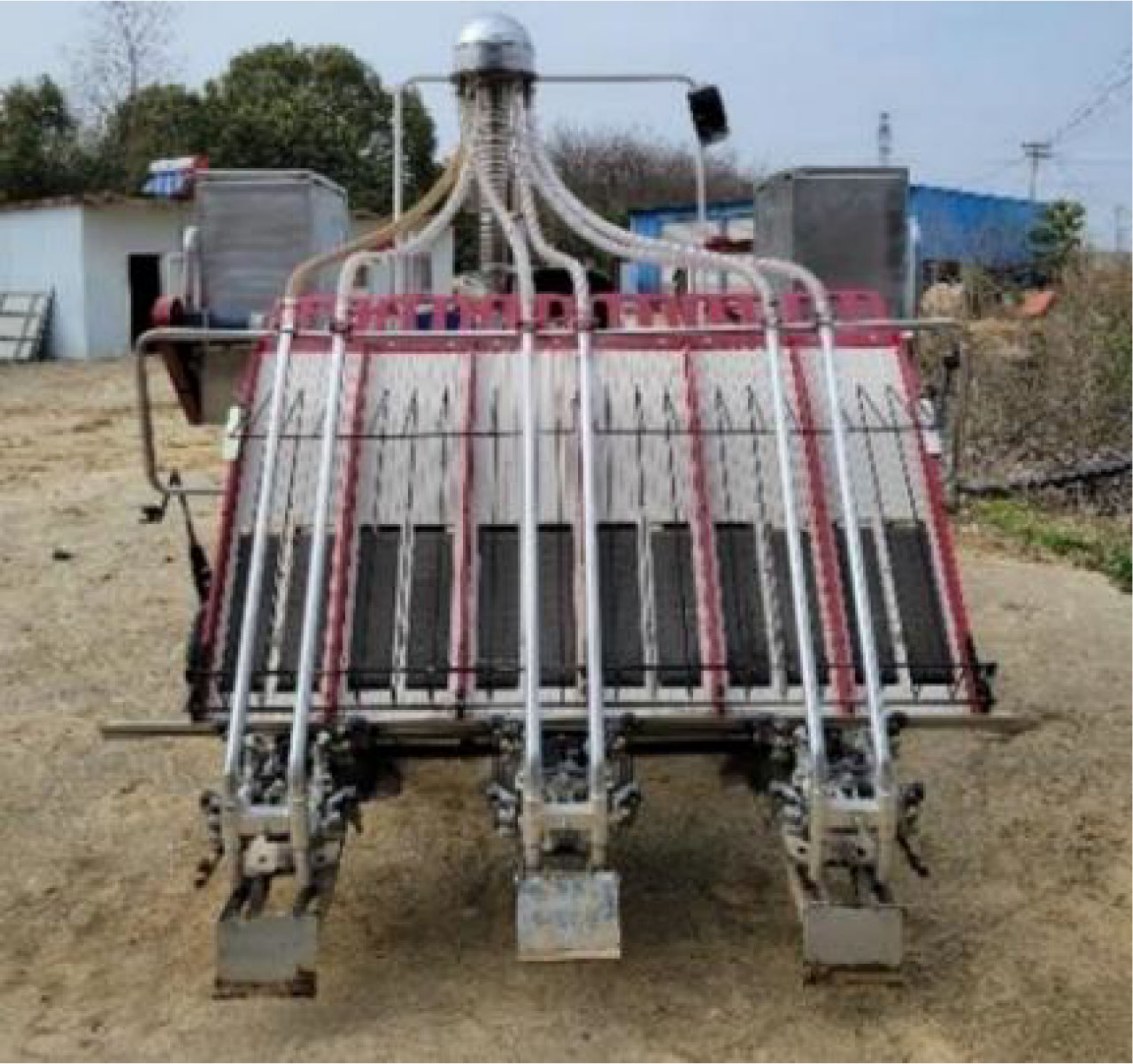	32	2.35
5	Mechanical fertilizer discharge + air-assisted	Suzhou Jofae Agricultural Machinery Co., Ltd.	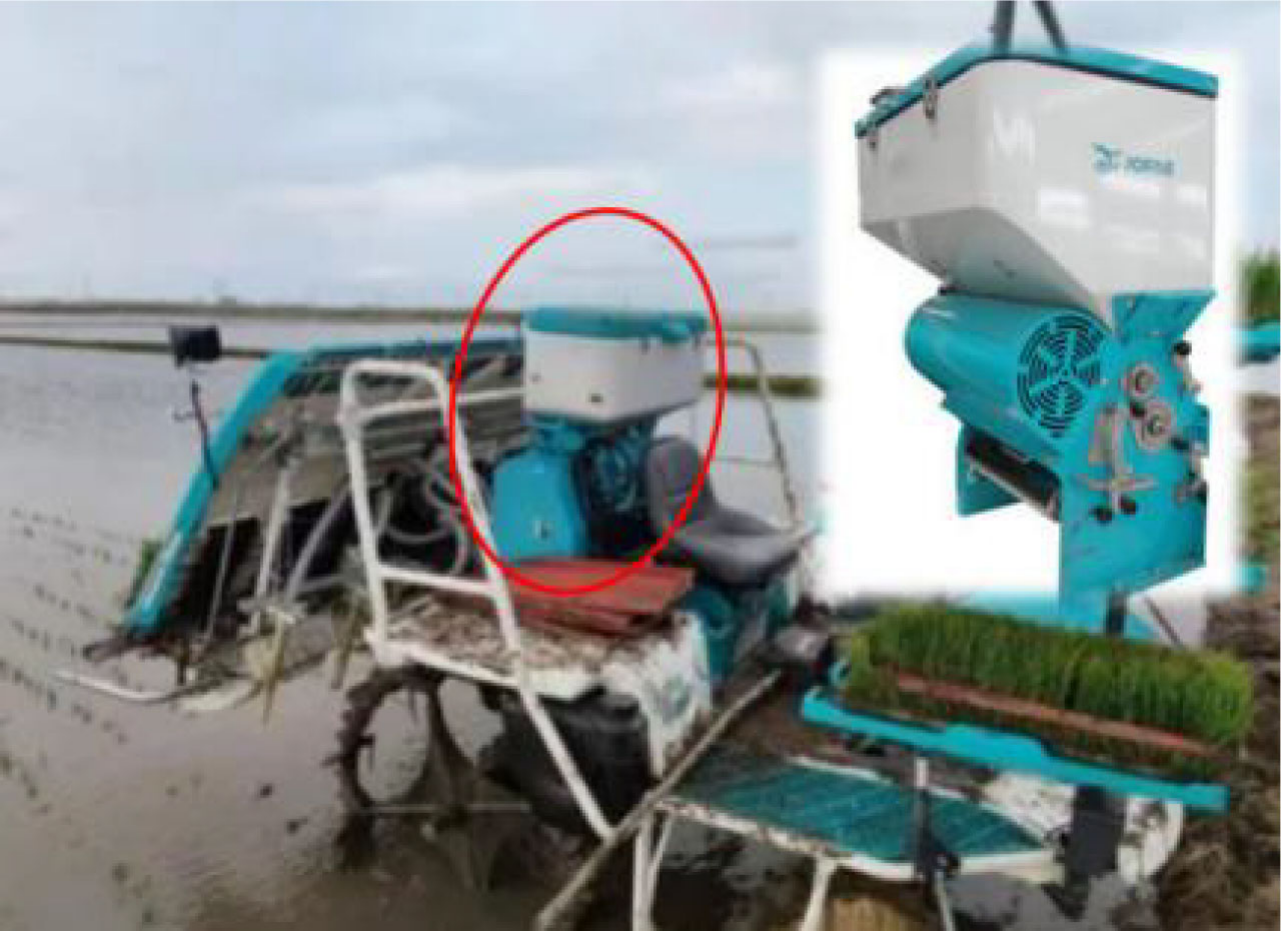 Jofae 2F1-1.8A(6F) Rice Side Deep Fertilizer Applicator	50	0.38

The coefficients of variation for air-blown fertilizer applicators in existing studies ranged from 3.7% to 5% (Cheng et al., 2022; Wang et al., 2024b; Yuan et al., 2022). For mechanical fertilizer discharge plus pneumatic-assisted delivery, they ranged from 1.49 to 3.61% (Cao et al., 2024; Zha et al., 2020). For purely mechanical fertilizer discharge were reported as 4.15 and 6.64–15.79% (Jiang et al., 2019; Wang, J. et al., 2023b). By adopting the OSMM for centrifugal distribution speed, we have achieved a row-to-row coefficient of variation below 7.0% under most operating speeds and fertilizer application rates. This not only surpasses the performance of existing purely mechanical fertilizer application devices, but also approaches the level of pneumatic conveying devices. Meanwhile, it meets the requirements of the National Standard (GB/T 20346.2–2022, CV<13%) and international standard (ISO 5690-2:2019, CV<15%). Fertilizer discharge error was below 7.9%, which could satisfy the requirements of the Industry standard (NY/T 1003-2006) for fertilizer application. At the same time, the range of operating speed was broader, the cost was lower, and the adaptability was stronger.

The lack of adaptability to irregular granular fertilizers may stem from the new calibration formula being based on spherical granular fertilizers, as the filling and driving layer coefficients of irregular granular fertilizers can differ substantially from those of spherical granular counterparts. This discrepancy leads to inadequate control accuracy for irregular granular fertilizers, necessitating further investigation to enhance the universality of the adaptive fertilizer application control system. Second, there is still room for optimizing the power control of the current fertilizer applicator; for instance, replacing the motor with one of higher energy density is expected to further reduce the weight. Finally, the closed-loop control structure remains the future direction. If a new sensor capable of effectively measuring fertilizer flow rate becomes available, it can be integrated into this study in the future, which is expected to achieve better fertilization accuracy.

## 5 Conclusion

In this study, the design of the mechanical fertilizer discharge+ mechanical centrifugal distribution reduced the mass of the entire fertilizer applicator to 13 kg. This represents a 16–40% reduction compared to the mass of conventional fertilizer applicators used within paddy field cultivation available in the market. The fertilizer applicator unit requires only two 150 W motors for power and lacks a repetitive mechanical structure; therefore, a considerable decrease in manufacturing cost can be expected.

A novel control system was also developed in the study. The new control system comprises an adaptive fertilizer application control module and an optimal speed-matched centrifuge distribution module. The adaptive fertilization control module adds bulk density to the calibration formula, and the bench test results indicate that the adaptive fertilization module can adapt to the quantitative discharge of different fertilizers. The optimal speed-matching module can accurately adjust the speed of the distributor according to the amount of fertilizer discharged, simultaneously saving electricity and reducing the collision of fertilizer particles.

Field trials showed that the individual row CV decreased to less than 7.0% across most operating speeds, while the fertilizer discharge error was below 7.9%. The operating results were significantly optimized compared with those without the control system. The dynamic and static test results exhibited no significant differences in the coefficients of variation between rows, indicating that the side-depth fertilizer application system was effectively adapted.

## Data Availability

The original contributions presented in the study are included in the article/supplementary material. Further inquiries can be directed to the corresponding author.
